# Isolation and characterization of new phenolic siderophores with antimicrobial properties from *Pseudomonas* sp. UIAU-6B

**DOI:** 10.3762/bjoc.17.156

**Published:** 2021-09-13

**Authors:** Emmanuel Tope Oluwabusola, Olusoji O Adebisi, Fernando Reyes, Kojo S Acquah, Mercedes De La Cruz, Larry L Mweetwa, Joy E Rajakulendran, Digby F Warner, Deng Hai, Rainer Ebel, Marcel Jaspars

**Affiliations:** 1 Marine Biodiscovery Centre, Department of Chemistry, University of Aberdeen, Scotland, UK; 2 Department of Microbiology, Faculty of Life Sciences, University of Ilorin, Kwara State, Ilorin, Nigeria; 3 Fundación MEDINA, Centro de Excelencia en Investigación de Medicamentos Innovadores en Andalucía, Avenida del Conocimiento 34, Parque Tecnoloógico de Ciencias de la Salud, E-18016 Granada, Spain; 4 Wellcome Centre for Infectious Diseases Research in Africa, University of Cape Town, Rondebosch, 7701, South Africa

**Keywords:** *Mycobacterium tuberculosis*, Phenolic siderophores, pseudomonine, *Pseudomonas* sp., vancomycin-sensitive *Enterococcus faecium*

## Abstract

Five new phenolic siderophores **1**–**5** were isolated from the organic extract of a culture broth in a modified SGG medium of *Pseudomonas* sp. UIAU-6B, obtained from sediments collected from the Oyun river in North Central Nigeria. The structure of the new compounds, pseudomonin A–C (**1**–**3**) and pseudomobactin A and B (**4** and **5**) isolated alongside two known compounds, pseudomonine (**6**) and salicylic acid (**7**), were elucidated based on high-resolution mass spectrometry, 1D and 2D NMR analyses. The absolute configuration of the threonine residue in compounds **1**–**5** was determined by Marfey analysis. The antimicrobial evaluation of compound **4** exhibited the most potent activity against vancomycin-sensitive *Enterococcus faecium* VS144754, followed by **3** and **5**, with MIC values ranging from 8 to 32 µg/mL. Compounds **2** and **3** exhibited moderate activity against *Mycobacterium tuberculosis* H37Rv, with MIC values of 7.8 and 15.6 µg/mL, respectively. Plausible biosynthetic hypotheses toward the new compounds **1**–**5** were proposed.

## Introduction

The introduction of antimicrobial drugs in the mid-20th century has had an unprecedented positive impact on human health, but the current threat of multiple drug resistance may well roll back all these past achievements [1]. One way out of this is an extensive search for novel bioactive natural products from microbial sources [2–3].

There is focus on the genus *Pseudomonas* for scientific research due to its widespread distribution in water, soil, and extreme habitats, including the exceptional ability to colonize the rhizosphere of host plants, serving as a microbial biocontrol [4–5]. This genus, belonging to the Gram-negative proteobacteria is diverse in nature and possesses unique environmental adaptability which shows in their versatile metabolism [6–8]. They are prolific producers of natural products with broad-spectrum biological functions which include antifungal [9–11] and antibacterial activities [12–14]. Natural product scaffolds isolated from species of this genus have contributed immensely as leads to drug discovery and development [15]. For instance, clofazimine [16], the antimycobacterial agent used for the treatment of leprosy and inflammation was inspired by the natural product phenazine [17–18]. Also, pseudomononic acid (mupirocin) isolated from *Pseudomonas fluorescens* by Fuller and co-workers in 1971 was discovered to possess novel antibacterial activities against 310 clinical isolates of *Staphylococcus aureus* with MIC values ranging from 0.06 to 0.25 µg/mL. Mupirocin was later developed for topical usage [19–22].

Several siderophores of hydroxamate, catecholate or phenolic scaffolds such as cepabactin [23], pyochelin [24–25], pyoverdines [26], vulnibactin [27], and pseudomonine [28] are produced under iron deficiency by various *Pseudomonas* species to acquire iron which is essential for cell metabolism and growth [29–30]. Studies show that siderophore–antibiotic complexes may be used as a Trojan horse strategy in which the antibiotics utilize the iron-siderophore transporting system as a cellular entry gateway [31–32]. Thus far, a few *Pseudomonas-*derived siderophores including ferrocins [33], thioquinolobactin [34], and (+)-(*S*)-dihydroaeruginioc acid [35] have been reported as active antimicrobial agents.

Our continued exploration of new bioactive compounds from microbial sources led to the isolation of *Pseudomonas* sp. UIAU-6B from sediments collected from the Oyun river in North Central Nigeria. Herein, we report the isolation and structure elucidation of seven secondary metabolites including five new (**1**–**5**), and two known phenolic siderophores (**6** and **7**) [28,36–37], together with their antimicrobial activities against vancomycin-sensitive *Enterococcus faecium* VS144754 and *Mycobacterium tuberculosis* strain H37Rv. A plausible biosynthetic hypothesis is proposed towards the new compounds **1**–**5**.

## Results and Discussion

**Figure 1:**
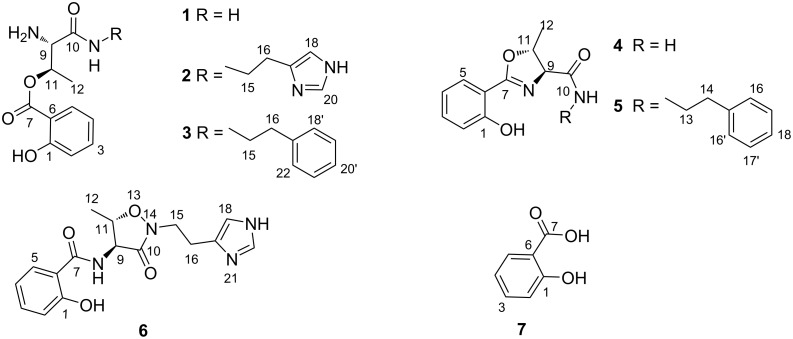
Structures of the new phenolic siderophores **1**–**5**, pseudomonine (**6**), and salicylic acid (**7**).

The preliminary LC–MS analysis of the methanolic extract from a small-scale culture of *Pseudomonas* sp. UIAU-6B in modified SGG medium indicated the presence of some interesting peaks with molecular ions which gave no hits when they were searched in natural product databases (Antibase). At the same time, the ^1^H NMR fingerprints suggested the presence of interesting aromatic compounds and led to a decision to undertake large-scale fermentation. The total crude extract (8.2 g) was subsequently subjected to a combination of fractionations (Kupchan partition and MPLC), and purification by reversed-phase semipreparative HPLC to obtain five new secondary metabolites **1**–**5** including pseudomonin A–C (**1**–**3**) and pseudomobactins A and B (**4** and **5**). The two known compounds, pseudomonine (**6**) and salicylic acid (**7**) were also isolated as the major constituents of the extracts and their structures were determined by analysis of HRESIMS, 1D and 2D NMR data (see Figure 1 and Supporting Information File 1 for details) which were comparable with those reported in the literature [28,36–37].

Pseudomonin A (**1**) was isolated as a yellowish compound. The molecular formula of C_11_H_15_O_4_N_2_ with 6 degrees of unsaturation was established by high-resolution ESI-Orbitrap-MS that revealed a molecular ion at *m*/*z* 237.0874 (calcd for C_11_H_13_N_2_O_4_^−^, 237.0880). A thorough analysis of ^1^H and 2D NMR data (see Table 1 and Supporting Information File 1) showed four aromatic methines, two aliphatic methines, one methyl, and four quaternary carbons signals at δ_C_ 162.9 (C-1), 113.2 (C-6), δ_C_ 170.0.0 (C-7), and δ_C_ 169.4 (C-10).

The downfield methine proton signals at δ_H_ 6.96 (d, *J* = 8.7, 1.8 Hz, H-2), 7.52 (td, *J* = 8.7, 7.2, 1.8 Hz, H-3), 6.94 (td, *J* = 8.7, 1.8 Hz, H-4), and δ_H_ 7.99 (dd, *J* = 8.1, 1.8 Hz, H-5) were consistent with a 1,2-disubstituted aromatic benzene ring of a salicylic acid unit, confirmed by long-range correlations observed in the HMBC spectrum from H-2, H-4 to C-6 (δ_C_ 113.2), and H-5 to the quaternary carbon C-7 (δ_C_ 170.0). On the other hand, the COSY spectrum confirmed the presence of a CH_3_–CH–CH spin system (H_3_-12, H-11, and H-9) reminiscent of a threonine unit. Remarkably, this unit was found to be attached in “reverse” fashion compared to the isoxazolidinone unit in compound **6**. The connection via an ester bond of the oxygenated sp^3^ methine C-11 (δ_C_ 71.1) with the carbonyl group of salicylic acid (C-7) was supported by a HMBC cross-peak from H-11 (δ_H_ 5.61) to C-7 (δ_C_ 170.0), and thus confirmed the structure of pseudomonin A (**1**).

**Figure 2:**
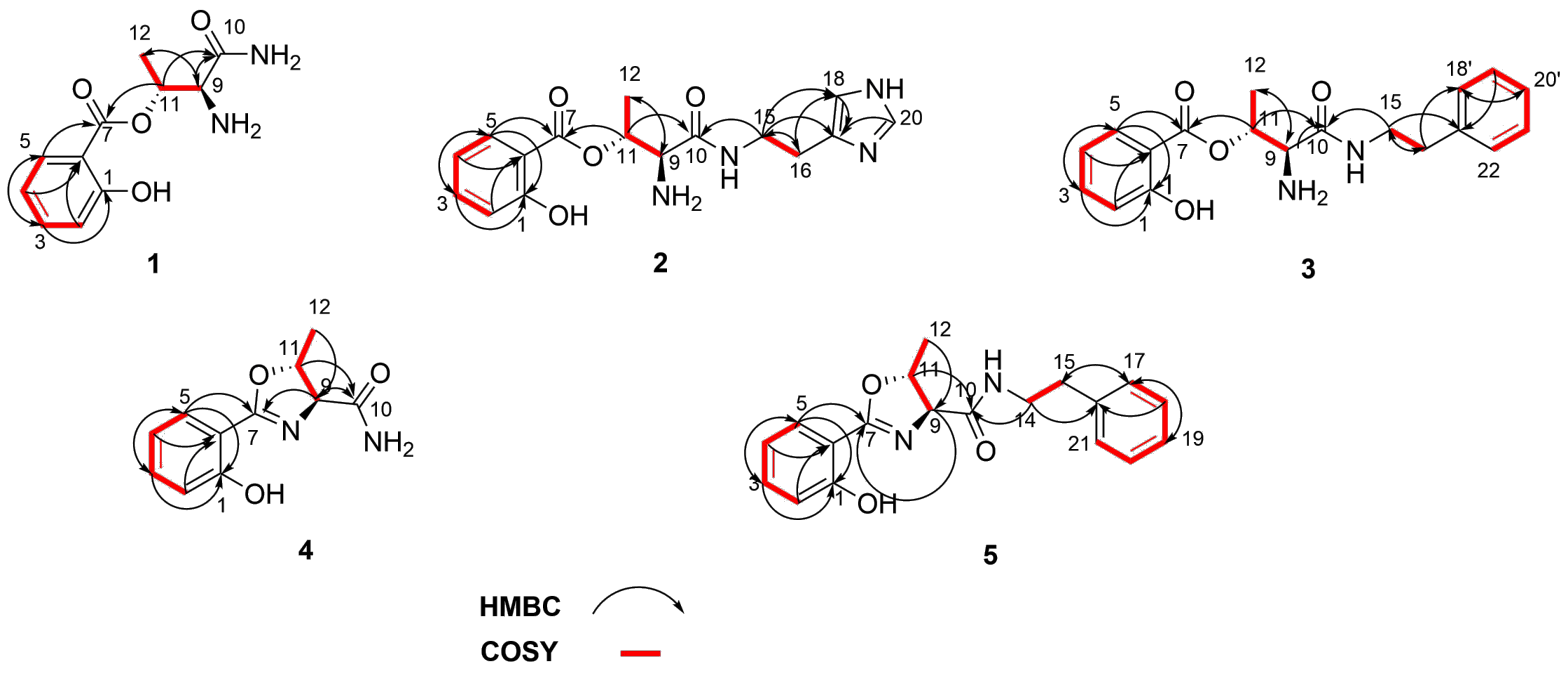
Key HMBC and ^1^H-^1^H COSY correlations.

Pseudomonin B (**2**) was isolated as a yellowish solid whose HRESI mass spectrum gave a molecular ion at *m*/*z* 333.1561 [M + H]^+^ that corresponded to a molecular formula of C_16_H_20_N_4_O_4_ (calcd for C_16_H_21_N_4_O_4_^+^, 333.1557) with 9 degrees of unsaturation. The detailed analysis of ^1^H and 2D NMR spectra (see Table 1 and Supporting Information File 1) suggested the presence of both, the salicylic acid (H-2/H-3/H-4/H-5) and the linear threonine subunits found in **1**, which were supported by the HMBC correlations shown in Figure 2. The major difference in comparison with compound **1** was the presence of a histamine subunit (H-15 to H-20), whose identity was established by COSY correlations observed between H-15 and H-16 and the key HMBC correlations depicted in Figure 2.

**Table 1:** ^1^H and ^13^C NMR data of pseudomonin A–C (**1**–**3**).^a^

	compound **1**		compound **2**		compound **3**	
position	δ_C_, type	δ_H_ (*J* in Hz)	δ_C_, type	δ_H_ ( *J* in Hz)	δ_C_, type	δ_H_ (*J* in Hz)

1	162.9, C		162.8, C		162.8, C	
2	118.2, CH	6.96, dd (8.7, 1.8)	118.4, CH	6.96, d (8.7)	118.4, CH	6.98, m
3	137.2, CH	7.52, td (8.7, 1.8)	137.5, CH	7.52, td (8.7, 1.7)	137.4, CH	7.54, td (8.7, 1.8)
4	120.2, CH	6.94, td (8.7, 1.8)	120.6, CH	6.94, dd (8.7, 1.7)	120.5, CH	6.95, m
5	131.6, CH	7.99, dd (8.7, 1.8)	131.7, CH	7.95, dd (8.7, 1.7)	131.7, CH	7.98, dd (8.7, 1.8)
6	113.2, C		113.1, C		113.2, C	
7	170.0, C		169.9, C		170.0, C	
9	57.6, CH	4.20, d (6.5)	57.7, CH	4.21, d (6.5)	57.8, CH	4.13, d (6.5)
10	169.4, C		167.7, C		167.2, C	
11	71.1, CH	5.67, qd (6.6, 6.5)	71.0, CH	5.61, qd (6.6, 6.5)	71.3, CH	5.53, dq (6.5, 6.5)
12	16.7, CH_3_	1.54, d (6.6)	17.0, CH_3_	1.49, d (6.6)	16.9, CH_3_	1.43, d (6.5)
15			39.3, CH_2_	3.62, dt (13.8, 6.8); 3.52, dt (13.8, 6.8)	42.1, CH_2_	3.44, dt (13.7, 7.2) 3.64, dt (13.7, 7.2)
16			25.3, CH_2_	2.92, m	36.1, CH_2_	2.79, m
17			132.5, C		140.0, C	
18			117.5 CH	7.33, d (0.6)		
20			135.0, CH	8.73, d (0.6)		
18’/22					129.5, CH	7.16, m
19’/21					129.6, CH	7.22, m
20’					127.5, CH	7.14, m

^a^Spectra acquired at 600 MHz in CD_3_OD for ^1^H NMR and at 150 MHz in CD_3_OD for ^13^C NMR.

Pseudomonin C (**3**) was isolated as white amorphous solid. Its molecular formula of C_19_H_22_N_2_O_4_ was deduced from the analysis of its HRESI mass spectrum (*m*/*z* 343.1653 [M + H]^+^, calcd for C_19_H_23_N_2_O_4_^+^, 343.1652) accounting for ten degrees of unsaturation. A prominent peak at *m*/*z* 325.1551 [(M + H − OH]^+^ (calcd for C_19_H_21_N_2_O_3_^+^, 325.1547) due to a facile loss of H_2_O from the molecular ion was observed. A thorough inspection of 1D and 2D spectra (see Table 1 and Supporting Information File 1) confirmed the resonance signals characteristic of both, the salicylic acid (H-2 to H-5) and the linear threonine (H-9 to H_3_-12) conjugate present in compounds **1** and **2**, and also displayed a new set of signals compatible with the replacement of the histamine unit in **2** with a phenylethylamine moiety (H-15 to H-22). This was supported by the COSY and HMBC correlations shown in Figure 2, and thus confirmed the structure of pseudomonin C (**3**).

Pseudomobactin A (**4**) was obtained as a reddish solid. The HRESI mass spectrum revealed a molecular ion at *m*/*z* 221.0923 ([M + H]^+^, calcd for C_11_H_13_N_2_O_3_^+^, 221.0921) corresponding to a molecular formula of C_11_H_12_N_2_O_3_ with seven degrees of unsaturation. The integration of the ^1^H NMR spectrum (see Table 2 and Supporting Information File 1) identified two spin systems consistent with the presence of the 1,2-disubstituted aromatic ring of salicylic acid in compounds **1**–**3** and a threonine unit (H-9 to H-12) that differed from compounds **1**–**3** due to the observed deshielded chemical shifts of the methine carbons C-9 (δ_C_ 75.5) and C-12 (δ_C_ 21.4), suggestive of the presence of an oxazoline ring similar to that found in vulnibactin [27]. The key HMBC cross-peaks (Figure 2 and Supporting Information File 1) from H-9 (δ_H_ 4.46, *J* = 7.3 Hz, d) and H-11(δ_H_ 4.90, qd, *J* = 6.3, 7.3 Hz) to carboxamide C-10 (δ_C_ 175.6) and C-7 (δ_C_ 167.8), confirmed the proposed structure which we named pseudomobactin A.

**Table 2:** ^1^H and ^13^C NMR data of pseudomobactin A (**4**) and B (**5**).^a^

	compound **4**		compound **5**	
position	δ_C_, type	δ_H_, (*J* in Hz)	δ_C_, type	δ_H_, (*J* in Hz)

1	161.1, C		161.1, C	
2	117.7, CH	6.96, d (8.3)	117.7, CH	6.99, d (8.3)
3	135.0, CH	7.41, td (8.3, 1.7)	135.1, CH	7.43, td (8.3, 1.6)
4	119.9, CH	6.90, td (7.4, 1.7)	120.0, CH	6.92, t (7.4, 1.7)
5	129.5, CH	7.68, dd(7.4, 1.7)	129.5, CH	7.67, dd (7.4, 1.7)
6	111.6, C		111.6, C	
7	167.8, C		168.0, C	
9	75.5, CH	4.46, d (7.3)	75.8, CH	4.38, d (7.3)
10	175.6, C		172.9, C	
11	80.6, CH	4.90, qd (6.3, 7.3)	80.6, CH	4.78, dq (6.3, 7.3)
12	21.4, CH_3_	1.57, d (6.3)	21.3, CH_3_	1.51, d (6.3)
14			41.9, CH_2_	3.44, dt (13.6, 7.2), 3.51, dt (13.6, 7.2)
15			36.3, CH_2_	2.82, t (7.2)
16			140.2, C	
17/21			129.9, CH	7.20, m
18/20			129.5, CH	7.22, m
19			127.3, CH	7.15, m

^a^Spectra acquired at 600 MHz in CD_3_OD for ^1^H NMR and at 150 MHz in CD_3_OD for ^13^C NMR.

Compound **5** was obtained as a reddish solid. The HRESIMS gave a prominent molecular ion at *m*/*z* 325.1550 ([M + H]^+^ corresponding to a molecular formula of C_19_H_20_N_2_O_3_ with eleven degrees of unsaturation. The information afforded by integrating the signals of the ^1^H NMR and the full 2D spectra (Table 2 and Supporting Information File 1) of compound **5** showed resonance signals consistent with compound **4** connected to the same 2-phenylethylamine moiety present in **3** (H-14 to H-21). This was supported by HMBC cross-peaks (Figure 2) between the diastereotopic protons H_2_-14 and carboxamide C-10 (δ_C_ 172.9) and the quaternary carbon C-16 (δ_C_ 140.2), between H_2_-15 and H-18/20 to the quaternary carbon C-16, and a strong correlation from H-15 and H-19 to C-17/20 (δ_C_ 129.9). The new oxazoline derivative **5** was named pseudomonbactin B.

The absolute configuration of the threonine residue in compounds **1**–**5** was assigned as ʟ, based on the derivatization with Marfey’s reagent of compound **2** and **4** hydrolysates, which represented the two distinctive structural scaffolds and chemical shifts followed by HPLC analysis of the derivatized amino acid residuals in the hydrolysate and threonine standards (see Supporting Information File 1).

**Figure 3:**
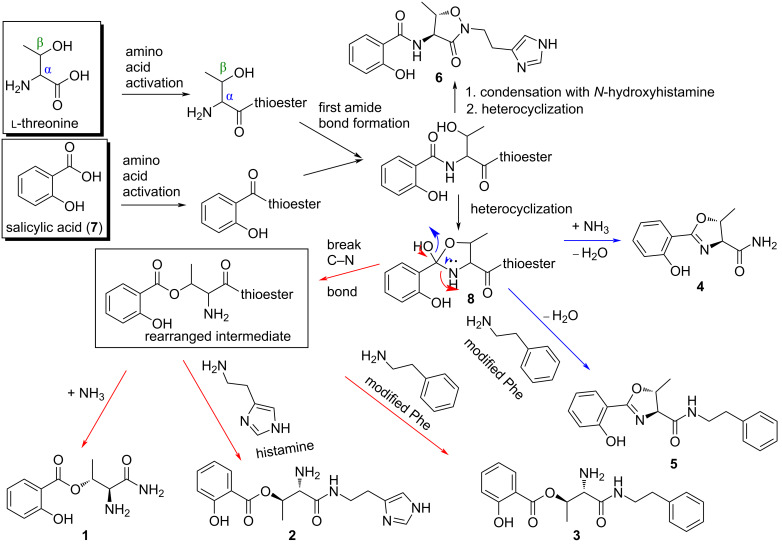
Plausible biosynthetic hypotheses of compounds **1**–**5**.

The biosynthesis hypotheses of compounds **1**–**5** were proposed to have originated as an extension of the reported pseudomonine (**6**) biosynthesis [37–40] via the salimethyloxazolinyl-thioester intermediate **8** (Figure 3). We speculated that compounds **1**–**3** occur through deviation of the salimethyloxazolinyl-thioester intermediate **8** from the assembly line via an unusually facile C–N-bond opening of the ring to generate an ester bond, and followed directly by amination (+ NH_3_), the addition of histamine and phenethylamine units to form compounds **1**, **2**, and **3**, respectively (Figure 3). Biochemical studies show that histamine and phenethylamine moieties were produced from histidine and phenylalanine substrates by a decarboxylase enzyme [41–43]. Pseudomobactin A (**4**) was proposed logically to have formed through direct amination of the unstable salimethyloxazolinyl-thioester intermediate followed by dehydration [44]. The incorporation of the decarboxylated phenylalanine by a nucleophilic unit to the intermediate salimethyloxazolinyl-thioester resulted in the formation of compound **5** without a rearrangement to isooxazolidinone owing to lack of N–OH in the phenylalanine unit [40].

### Biological evaluation

Compounds **1**–**7** were evaluated for their antimicrobial activities against methicillin-resistant *Staphylococcus aureus* (MRSA), methicillin-sensitive *Staphylococcus aureus* (MSSA), *Escherichia coli*, *Acinetobacter Bahmani*, vancomycin-resistant *E. faecium VanA*15167*,* vancomycin-sensitive *E. faecium* VS144756, and *M. tuberculosis* H37Rv (see Table S8 in Supporting Information File 1). Compound **4** was the most potent against Vancomycin-sensitive *E. faecium* VS144754, with a MIC value in a range of 8–16 µg/mL, followed by compounds **3** and **5**, which showed MIC values of 16 and 32 µg/mL, respectively. Additionally, compounds **2** and **3** exhibited moderate antitubercular activity with MICs of 7.8 and 15.6 µg/mL, respectively. Besides, compound **5** showed low activity (MIC of 125 µg/mL) against the latter pathogen. None of the compounds inhibited the growth of *E. coli, A. baumannii*, MRSA, MSSA, or *E. faecium VanA*15167 at the highest concentration (64 µg/mL) tested (see Supporting Information File 1).

## Conclusion

In summary, we have isolated seven natural products, including five new phenolic siderophores (**1**–**5**) that have not been reported in the literature and their biosynthetic pathways were proposed as a rational extension of the previously reported metabolite, pseudomonine (**6**), one of the major constituents of the extract from the culture broth of *Pseudomonas* sp. UIAU-6B. The new compounds **1**–**5** are interesting chemical entities consisting of scaffolds with different functionalities that could be exploited for further biological investigation and structure–activity relationship (SAR) studies. To the best of our knowledge, the breakage of the C–N bond of the salimethyloxazolinyl-thioester intermediate leading to an ester bond that resulted in the formation of compounds **1**–**3** constitutes new plausible hypotheses not previously reported in natural product biosynthesis.

## Experimental

### General experimental procedures

IR spectra were acquired on a Perkin Elmer Spectrum Two FT-IR spectrometer equipped with an ATR diamond cell. Optical rotations were measured on an ADP 410 digital Polarimeter (Bellingham + Stanley Ltd, United Kingdom). NMR spectra were recorded on Bruker AVANCE III spectrometers at 500/600 MHz for ^1^H NMR and 125/150 MHz for ^13^C NMR operating with a helium-cooled cryoprobe and a liquid nitrogen ‘Prodigy’ cryoprobe, respectively. ^1^H and ^13^C chemical shifts were referenced to the residual non-deuterated solvent peak (3.31 and 49.1 ppm for CD_3_OD). The multiplicities of ^13^C NMR were determined by an edited heteronuclear single quantum coherence (HSQC) experiment. HRESIMS spectra were obtained using a Thermo Instruments MS system (LTQ XL/LTQ Orbitrap Discovery) coupled to a thermo instruments HPLC system (Accela PDA detector, Accela PDA autosampler, and Accela pump) or an Agilent 6540 in ESI-TOF MS coupled to an HPLC Agilent 1290 Infinity equipped with a diode array detector (DAD). Chromatographic fractionation was carried out on a Reveleris preparative flash system equipped with a reversed-phase Phenomenex strata^®^ C18-E cartridge (55 µm, 70 Å, 2 g/12 mL). HPLC purification was done with an Agilent HPLC apparatus (1200 series) equipped with a binary pump, diode array detector (G1315B), sunfire reversed-phase column C18 (5 µm, 10 × 250 mm), Phenomenex C18 (5 µm, 9.4 × 250 mm), or ACE C18 (5 µm, 9.4 × 250 mm) columns, and a mobile phase solvent gradient consisting of H_2_O (Milli-Q filter water 18 MΩ·cm, Millipore, Germany), 100% MeOH (Sigma-Aldrich, UK), and TFA (Sigma-Aldrich, UK).

### Bacterial isolation and identification

The bacterial strain was isolated from soil sediments collected from the Oyun river in North Central Nigeria (8.4799° N, 4.5418° W). The sediment samples were air-dried at room temperature for 48 h. Ten grams (dry weight equivalent) were suspended in 100 mL of sterile distilled water, vortexed at high speed, and 1 mL of diluent was serially diluted (10^−2^ and 10^−4^). Isolation of the bacterium (UIAU-6B) using a spread plate technique was performed by inoculating an 0.1 mL aliquot on pseudomonas agar base augmented with cephalosporin–fucidin–cetrimide-selective supplement (Pseudomonas–CFC agar SR0103, Oxoid Ltd., Reading, UK) and incubated at 28 °C for five days. The pure colony of the UIAU-6B strain was purified by repeated subculture on pseudomonas agar base plates. The strain was identified by analysis of the molecular DNA sequencing of its 16S rDNA and the sequence obtained was compared with the Genbank database using the BLAST search of the National Center for Biotechnology Information (NCBI) which confirmed it to be homologous by 99.06% in the phylogenetic tree as *Pseudomonas* sp. (see Figure S49A, in Supporting Information File 1).

### Fermentation

The small-scale culture was prepared by inoculating a stock culture of *Pseudomonas* UIAU-6B (2 mL) in a 1000 mL baffled Erlenmeyer flask containing 150 mL modified SGG medium (glycerol 10 g, corn steep powder 2.5 g, peptone 5 g, soluble starch 10 g, yeast extract 2 g, CaCO_3_ 3 g, NaCl 1 g, in 1 L H_2_O, pH adjusted to 7.3) [45]. The flask was left shaking at 160 rpm in a closed system shaker for ten days at 28 °C. Subsequently, 7.5 g of HP-20 resin were added to the fermentation cultures under sterile conditions and incubated for the next 7 h at the same shaking conditions. The broth cultures were filtered under vacuum and the HP-20 resin was extracted with methanol (200 mL) to obtain 1.2 g of crude extract from which a 0.5 mg/mL solution was prepared for preliminary LC–MS chemical profiling.

Large-scale cultures (6000 mL) were prepared by inoculating twenty 2000 mL baffled Erlenmeyer flasks containing 300 mL modified SGG medium with the seed culture of *Pseudomonas* UIAU-6B (50 mL). The seed culture (50 mL) was prepared by inoculating a 250 mL baffled Erlenmeyer flask containing a modified SGG medium with a stock culture of *Pseudomonas* UIAU-6B (2 mL) and growth for 3 days. The entire fermentation process employed similar conditions as the small-scale culture.

### Extraction and isolation

After the incubation, 15 g of HP-20 resin were added to each flask under sterile conditions and allowed to shake for 7 hours before the HP20 resin was filtered and subsequently extracted with methanol (3 × 500 mL) followed by ethyl acetate (3 × 300 mL). The combined organic extract was dried under reduced pressure, to yield 8.2 g of crude extract. The extract (8.2 g) was dissolved in H_2_O and CH_2_Cl_2_ (700 mL, 50% v/v) and placed in a separating funnel for 5 min to allow for proper separation of the constituent mixture between the aqueous (6.21 g) and organic phases (1.99 g) [46]. The dichloromethane fraction (1.99 g) was chromatographed on a reversed-phase preparative flash system consisting of a C-18 (KP-C18-HS™, 40 × 150 mm) stationary phase cartridge equipped with variable UV and ELSD detectors to monitor the run at 210, 230, and 254 nm) with the combination of isocratic and linear gradient solvent system (80–20% H_2_O/MeOH for 20 min followed by a linear gradient from 20% to 50% MeOH over 10 min, 50% MeOH for 5 min, 50% to 70% MeOH/H_2_O for 15 min, 70% MeOH for 5 min, 70% to 100% MeOH/H_2_O for 15 min and 100% MeOH over 20 min) at a flow rate of 10 mL/min. Four fractions were obtained: FD1 (0.8 g, *t*_R_ 8.2–12.0 min), FD2 (0.35 g, *t*_R_ 27–40 min), FD3 (0.24 g, *t*_R_ 41–57 min), and FD4 (0.45 g, *t*_R_ 58–70 min).

Fraction FD1 (0.8 g) was subjected to reversed-phase semipreparative HPLC on a Sunfire column C18 (5 µm, 10 × 250 mm) with a linear gradient of H_2_O/MeOH 80–20% over 30 min at a flow rate of 2 mL/min, yielding compounds **7** (70 mg, *t*_R_ 10 min), **6** (300 mg, *t*_R_ 15.1 min), and **1** (2.2 mg, *t*_R_ 23.4 min).

Fraction FD2 (0.35 g) was purified with the same HPLC solvent system and stationary phase as FD1 to obtain compound **4** (3.9 mg, *t*_R_ 22.2 min).

Fraction FD3 (0.35 g) was subjected to HPLC on a reversed-phase Phenomenex column C18 (5 µm, 10 × 250 mm) with a mobile phase gradient system of H_2_O/MeOH 80–20% and 0.01% TFA for 35 min at a flow rate of 2 mL/min to afford compound **2** (15.3 mg, *t*_R_ 19.1 min).

The final dichloromethane subfraction FD4 (0.45 g) was rechromatographed by reversed-phase HPLC using a Sunfire column C18 (5 µm, 10 × 250 mm) and a variable solvent system starting with H_2_O/MeOH 70:30 isocratic elution over 5 min, followed by a linear gradient to 100% MeOH over 20 min at a flow rate of 1.8 mL/min to yield compounds **3** (1.2 mg, *t*_R_ 16.8 min) and **5** (1.8 mg, *t*_R_ 18.9 min).

Pseudomonin A (**1**): yellowish oil; [α]_D_^25^ −9.5 (*c* 0.02, MeOH); UV (MeOH) λ_max_, nm (log ε): 276 (3.23); IR (cm^−1^) ν_max_: 3333, 1677 1660, 1538, 1493, 1203, 1138; NMR data, see Table 1; HRESIMS (*m*/*z*): [M − 1]^−^ calcd for C_11_H_13_N_2_O_4_, 237.0880; found, 237.0874, Δ = −2.53 ppm.

Pseudomonin B (**2**): yellowish oil; [α]_D_^25^ −13.3 (*c* 0.05, MeOH); UV (MeOH) λ_max_, nm (log ε): 297 (3.64); IR (cm^−1^) ν_max_: 3137, 1673, 1660; NMR data, see Table 1; HRESIMS (*m/z*): [M + H]^+^ calcd for C_16_H_21_O_4_N_4_, 333.1557; found 333.1561, Δ = 1.20 ppm.

Pseudomonin C (**3**): white amorphous solid; [α]_D_^25^ −45.6 (*c* 0.02, MeOH); UV (MeOH) λ_max_, nm (log ε): 299 (3.85); IR (cm^−1^) ν_max_: 3341, 2927, 1670, 1633, 1205; NMR data, see Table 1; HRESIMS (*m/z*): [M + H]^+^ calcd for C_19_H_23_N_2_O_4_, 343.1652; found, 343.1653, Δ = 2.01 ppm.

Pseudomobactin A (**4**): yellow amorphous solid; [α]_D_^25^ −17.7 (*c* 0.05, MeOH); UV (MeOH) λ_max_, nm (log ε): 260 (3.59), 302 (4.12); IR (cm^−1^) ν_max_: 3320, 2930, 1675, 1638; NMR data, see Table 2; HRESIMS (*m/z*): [M + H]^+^ calcd for C_11_H_13_N_2_O_3_, 221.0921; found, 221.0923, Δ = 0.90 ppm.

Pseudomobactin B (**5**): white amorphous solid; [α]_D_^25^ −28.8 (*c* 0.03, MeOH); UV (MeOH) λ_max_, nm (log ε): 267 (3.19); IR (cm^−1^) ν_max_: 3320, 2930, 1674, 1638, 1205; NMR data, see Table 2; HRESIMS (*m/z*): [M + H]^+^ calcd for C_19_H_21_N_2_O_3_, 325.1547; found, 325.1550, Δ = 0.92 ppm.

### Marfey’s analysis

Samples of compound **2** (150 µg) and **4** (150 µg) were dissolved separately in 2 M HCl (100 µL) and heated at 100 °C in sealed vials for 16 h. The hydrolysates were concentrated at 40 °C to dryness under a nitrogen stream. The hydrolysates were treated with 1 M NaHCO_3_ solution (20 µL) and 1% solution of ʟ-FDAA (Marfey's reagent, 1-fluoro-2-4-dinitrophenyl-5-ʟ-alanine amide) in acetone (40 µL). The reaction mixtures were gently heated at 40 °C for 1 h, after which the solutions were neutralized by the addition of 1 M HCl (20 µL), diluted with acetonitrile (100 µL), and filtered through a PTFE membrane filter (0.45 µm). The standard amino acids (ᴅ-Thr, ʟ-Thr, and ʟ-*allo*-Thr) were subjected to derivatization with ʟ-FDAA by using the same method previously described. For the HPLC analysis, mixtures of the standard amino acid derivatized solution (5 µL), compound **2** (5 µL) and **4** (5 µL) hydrolysates were analyzed by injecting into an HPLC Agilent 1260 Infinity instrument coupled with a Phenomenex C18 analytical column (5 µm, 4.6 × 150 mm) maintained at 40 °C. The mobile phase solvents comprised a mixture of A (100% water, 0.1% FA) and B (100% acetonitrile, 0.1% FA), with a linear gradient elution mode 10 to 65% CH_3_CN for 50 min and 65–100% CH_3_CN over 20 min at a flow rate of 1.0 mL/min and monitored by a diode array HPLC detector at 340 nm. The HPLC traces were aligned and visualized using data analysis as shown in Figure S47 (Supporting Information File 1). Retention times (min) for the derivatized (ʟ-FDAA) threonine standards and for the observed peaks in the HPLC trace of each ʟ-FDAA-derivatized hydrolysis product under the reported conditions were as follows: retention times of standards: ʟ-Thr: 19.62, ᴅ-Thr: 22.47, ʟ-*allo*-Thr: 19.83. Retention time (min) for the derivatized (ʟ-FDAA) threonine presents in **2**: ʟ-Thr: 19.63. Retention time (min) for the derivatized (ʟ-FDAA) threonine presents in **4**: ʟ-Thr: 19.64.

## Supporting Information

HRESIMS profiles and copies of NMR spectra for compounds **1**–**7** in CD_3_OD including the NMR table of **6** and **7** and copies of UV and IR of compounds **1**–**5**. Phylogenetic tree showing *Pseudomonad* sp. DNA sequence of the bacterial strain UIAU-6B. Chromatographic profiles of the compounds derivatized with ʟ-FDAA. Photo of *Pseudomonad* sp. growing on ISP2 an agar plate. Minimum inhibitory concentration of **1–7** for vancomycin-sensitive *Enterococcus faecium* VS144754 and *Mycobacterium tuberculosis* H37Rv. Experimental procedure for antimicrobial assays.

File 1 Additional analytical and experimental information.
