# Supplementary material for: Isolation and characterization of new phenolic siderophores with antimicrobial properties from Pseudomonas sp. UIAU-6B
Source: Beilstein J Org Chem. 2021 Sep 13;17:2390–8. doi: 10.3762/bjoc.17.156 (PMC8450953; doi:10.3762/bjoc.17.156)
Supplement: File 1 — Additional analytical and experimental information. [file Beilstein_J_Org_Chem-17-2390-s001.pdf]

BEILSTEIN JOURNAL OF ORGANIC CHEMISTRY

Supporting Information

for

Isolation and characterization of new phenolic siderophores with antimicrobial properties from *Pseudomonas* sp. UIAU-6B

Emmanuel T. Oluwabusola, Olusoji O. Adebisi, Fernando Reyes, Kojo S. Acquah, Mercedes De La Cruz, Larry L. Mweetwa, Joy E. Rajakulendran, Digby F. Warner, Deng Hai, Rainer Ebel and Marcel Jaspars

Beilstein J. Org. Chem. **2021,** *17,* 2390–2398. doi:10.3762/bjoc.17.156

Additional analytical and experimental information

License and Terms: This is a supporting information file under the terms of the Creative Commons Attribution License (https://creativecommons.org/licenses/by/4.0). Please note that the reuse, redistribution and reproduction in particular requires that the author(s) and source are credited and that individual graphics may be subject to special legal provisions.
The license is subject to the *Beilstein Journal of Organic Chemistry* terms and conditions: (https://www.beilstein-journals.org/bjoc/terms)

Table of contents

Figure S1-S2 (+)-HRESIMS and ^{1}H NMR spectra of **1** S3

Figure S3-S4 HSQC and COSY NMR spectra of **1**..S4

Figure S5-S6 HMBC of 1 and UV data **1**..S5

Figure S7-S8 IR data of **1** and HRESIMS spectrum of **2**..S6

Figures S9-S10 ^{1}H NMR and HSQC spectra of **2** S7

Figure S11-S12 COSY and HMBC spectra of **2** S8

Figure S13-S14 UV and IR data of **2** S9

Figure S15-S16 **(+)-**HRESIMS spectrum and ^{1}H NMR spectrum of **3** S10

Figure S17-S18 HSQC and COSY spectra of **3** S11

Figure S19-S20 HMBC spectrum and UV data of **3** S12

Figure S21-S22 IR of **3** and (+) HR-ESI-MS data of **4** S13

Figure S23-S24 ^{1}H NMR of **4** and HSQC spectra of **4** S14

Figure S25-S26 COSYand HMBC spectra of **4** S15

Figure S27-S28 UV and IR data of **4** S16

Figure S29-S30 (+)-HR-ESIMS and ^{1}H NMR spectra of **5** S17

Figure S31-S32 HSQC and COSY spectra of **5** S18

Figure S33-S34 HMBC spectrum and UV spectrum of **5** S19

Figure S35-S36 IR spectrum of **5** and (+)- HRESI-MS data of **6** S20

Figure S37-S38 ^{1}H NMR and ^{13}C spectrum of **6** S21

Figure S39-S40 HSQC and COSY spectra of **6** S22

Figure S41-S42 HMBC spectrum of **6** and (+)- HRESI-MS data of **7** S23

Figure S43-S44 ^{1}H NMR and HSQC spectra of **7** S24

Figure S45-S46 COSY and HMBC spectra of **7** S25

Figure S47-48 Chromatographic profiles of the compound (**2**) and (**4**) derivatized with L-FDAA and the standard amino acids with L-FDAA and DNA sequence of the bacterial strain UIAU-6B S26

Figure S49 (**A**) Phylogenetic tree showing *Pseudomonad* sp. and (**B**) Photo of *Pseudomonad* sp. growing on ISP2 an agar plate S27

Table S1-S2 NMR data consisting of COSY and HMBC for **1** and **2** S28

Table S3-S4 NMR data consisting of COSY and HMBC for **3** and **4** S29

Table S5-S6 NMR data consisting of COSY and HMBC for **5** and **6** S30

Table S7 NMR data consisting of COSY and HMBC for **5** and **6** S31

Experimental Procedure for antimicrobial assays S31

Table S8 Antimicrobial activity (MIC in µg/ mL) of compounds **1**-**7** S32

References S32

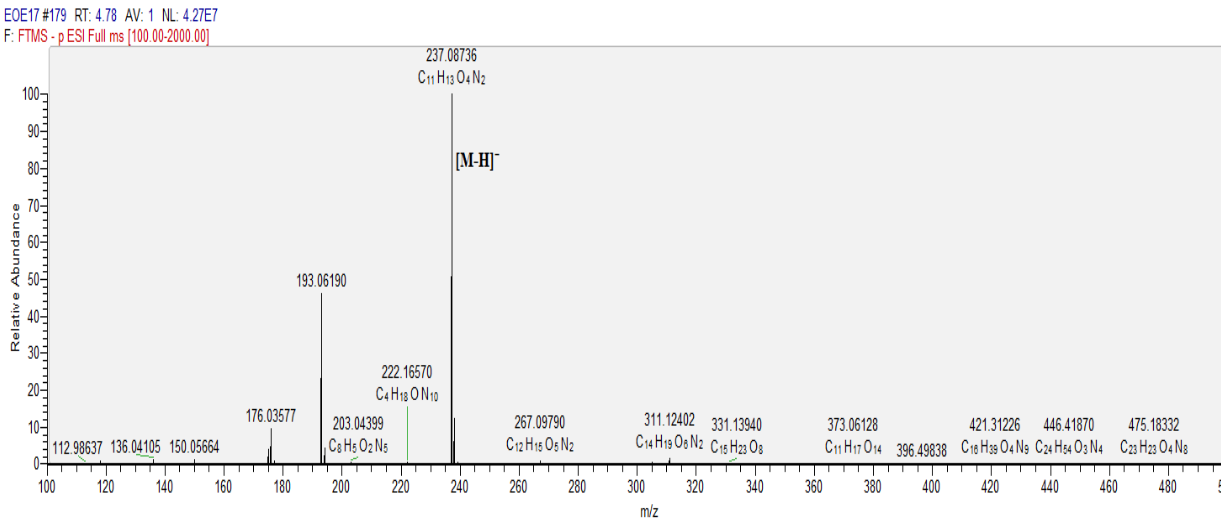

Figure S1. (+)-HR-ESIMS data of **1**

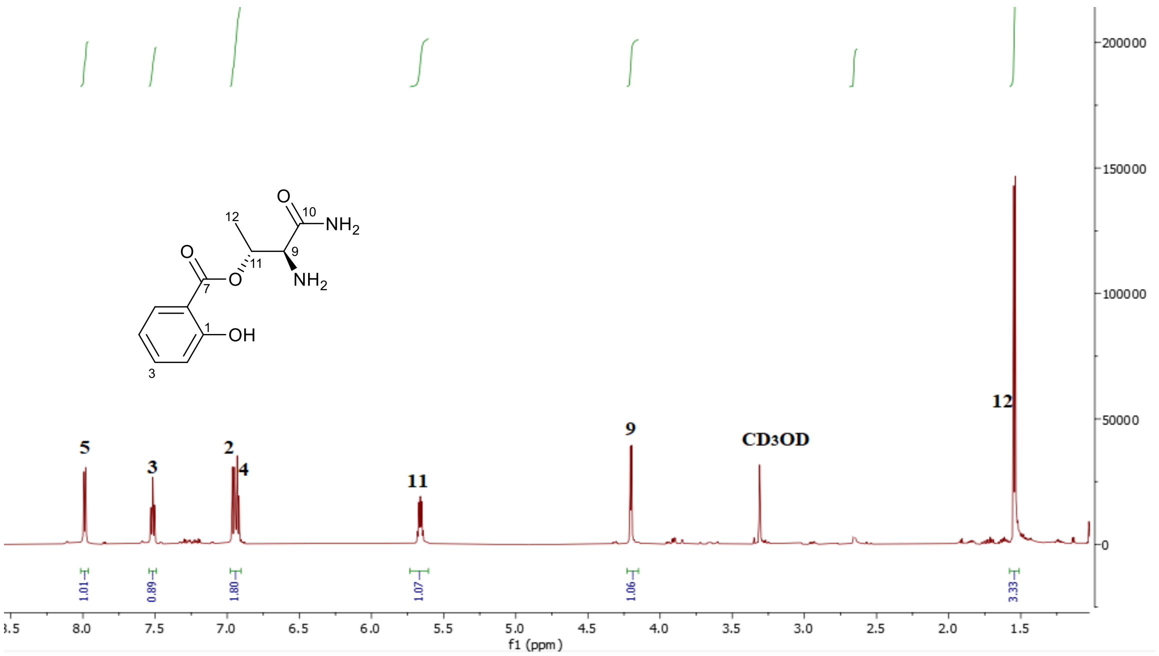

Figure S2. ^{1}H NMR spectrum of **1**

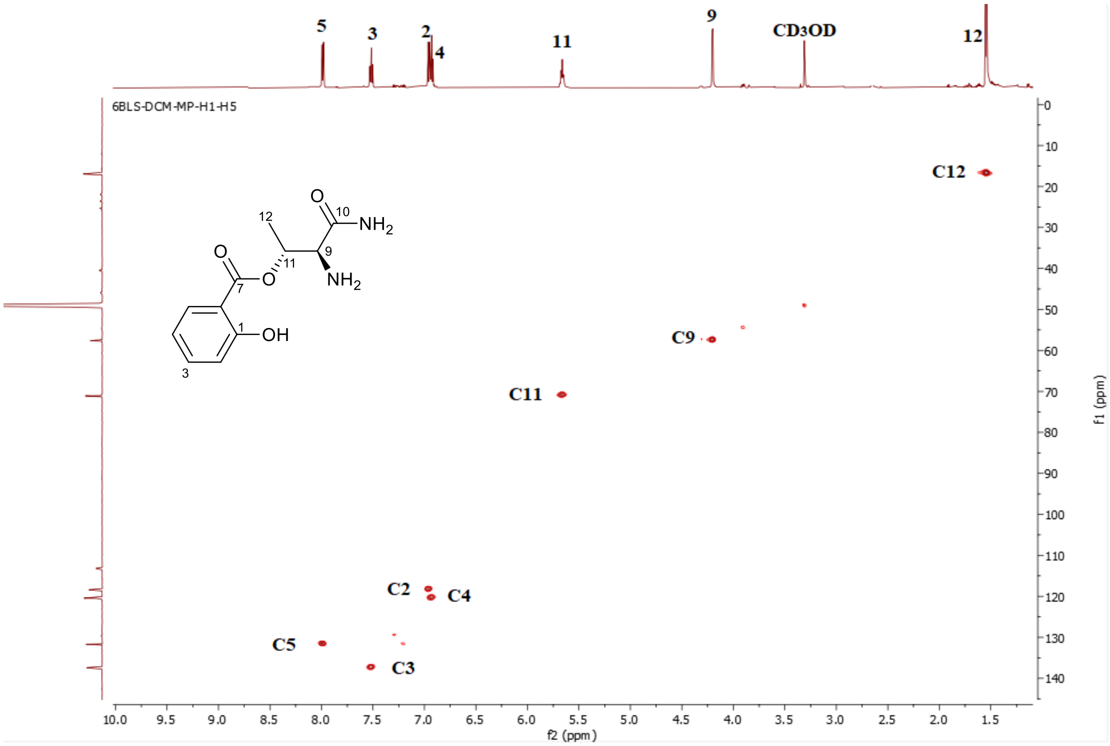

Figure S3. HSQC NMR spectrum of **1**

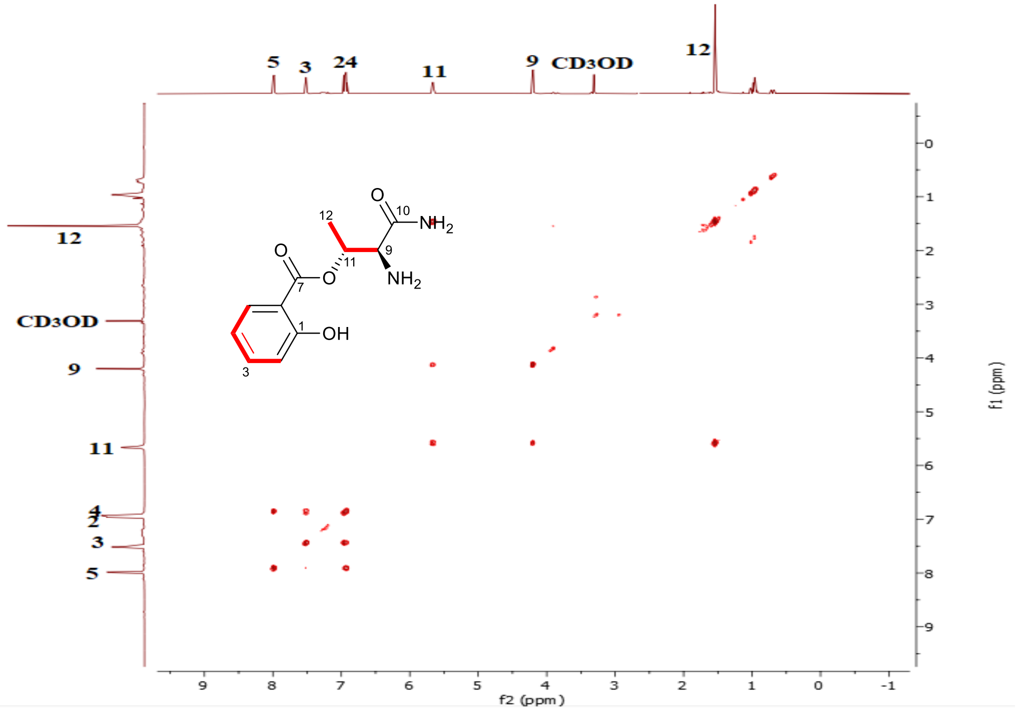

Figure S4. COSY NMR spectrum of **1**

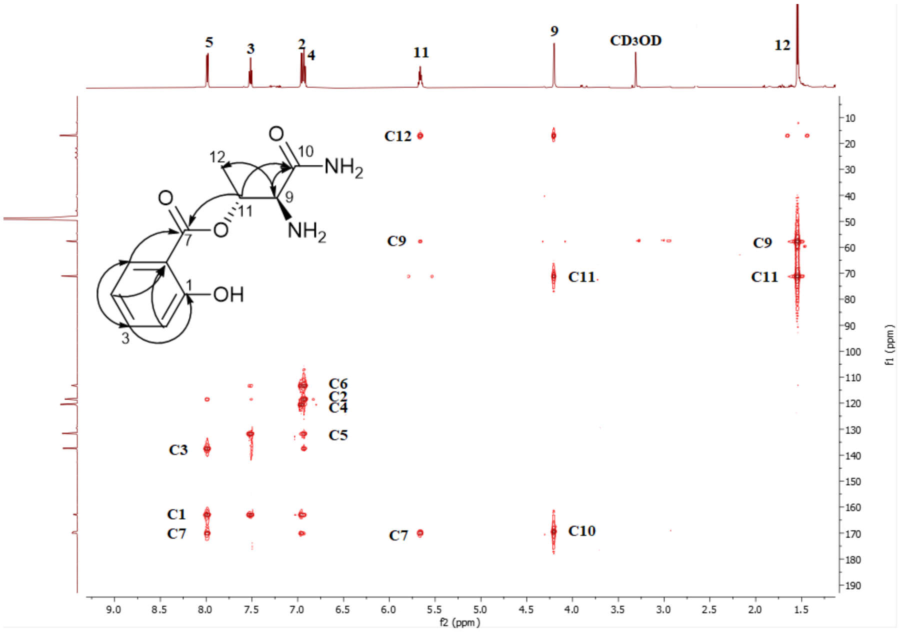

Figure S5. HMBC NMR spectrum of **1**

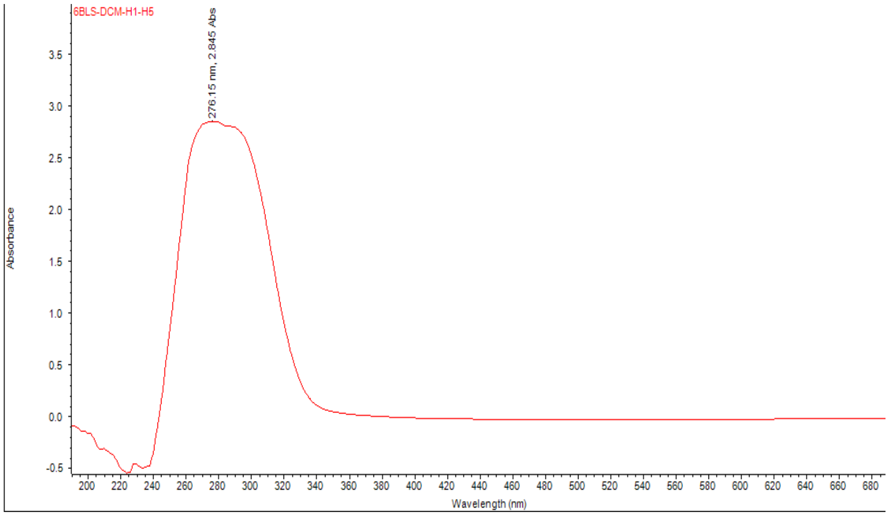

Figure S6. UV data of **1**

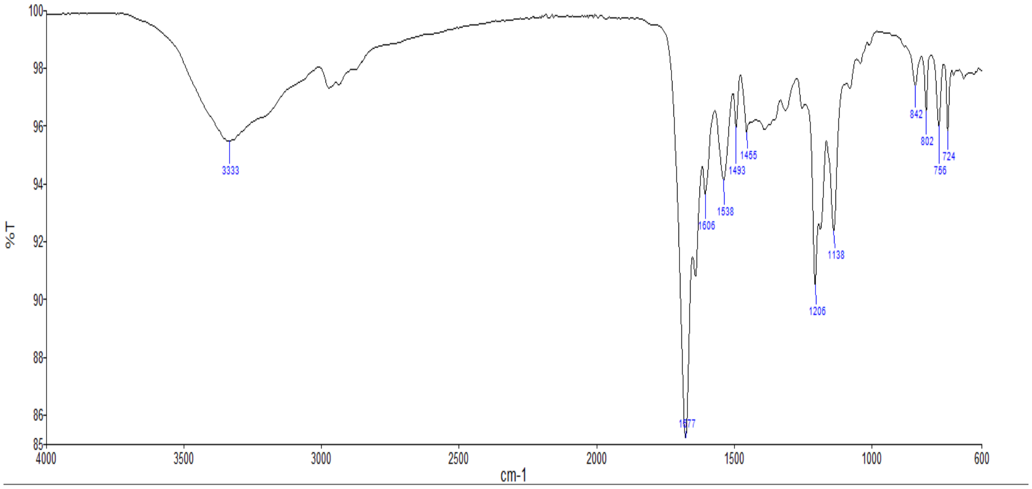

Figure S7. IR data of **1**

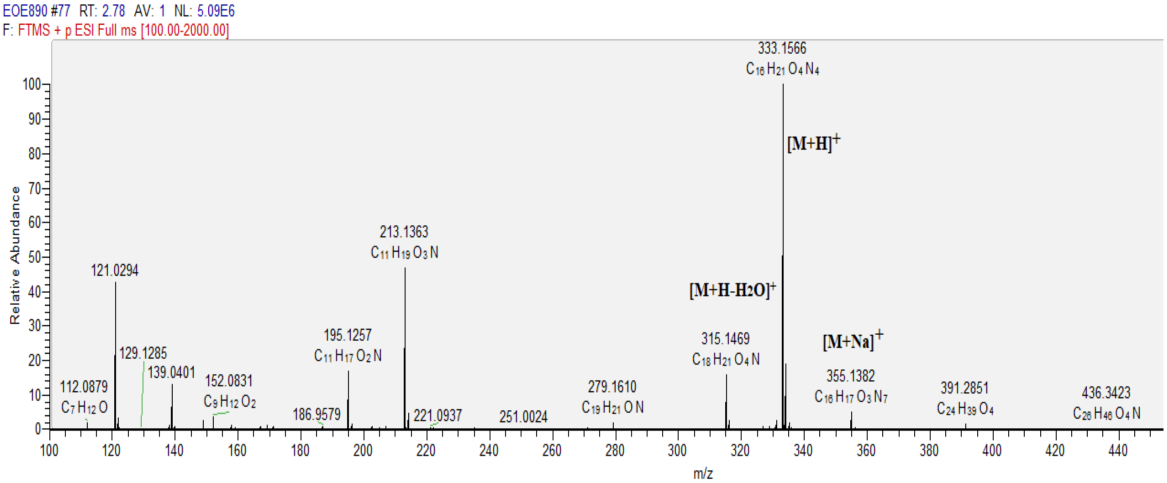

Figure S8. (+)-HR-ESIMS data of **2**

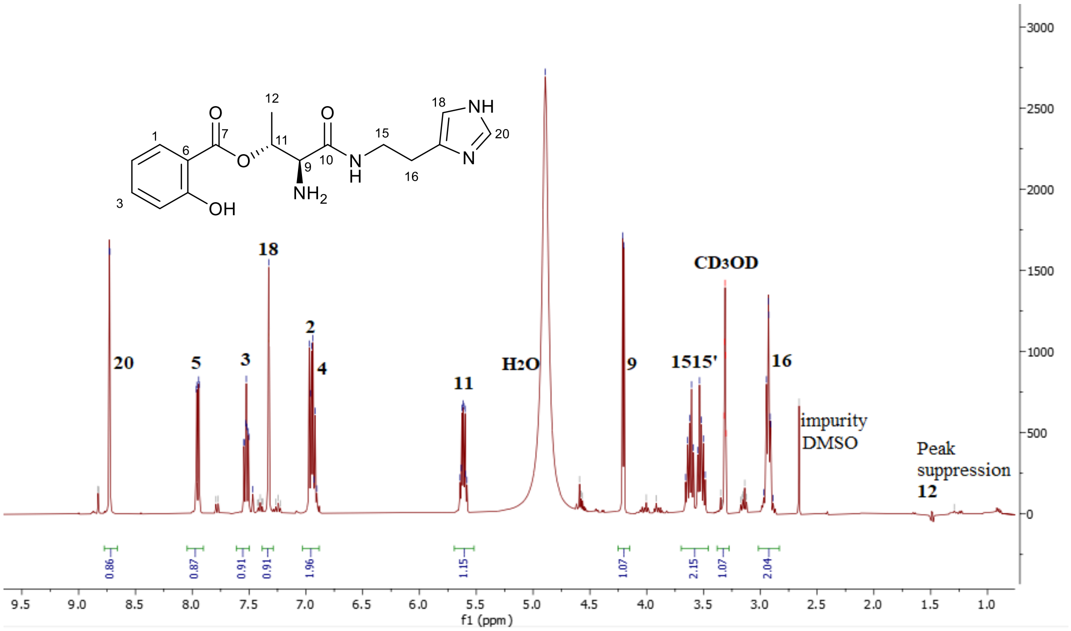

Figure S9. ^{1}H NMR spectrum of **2**

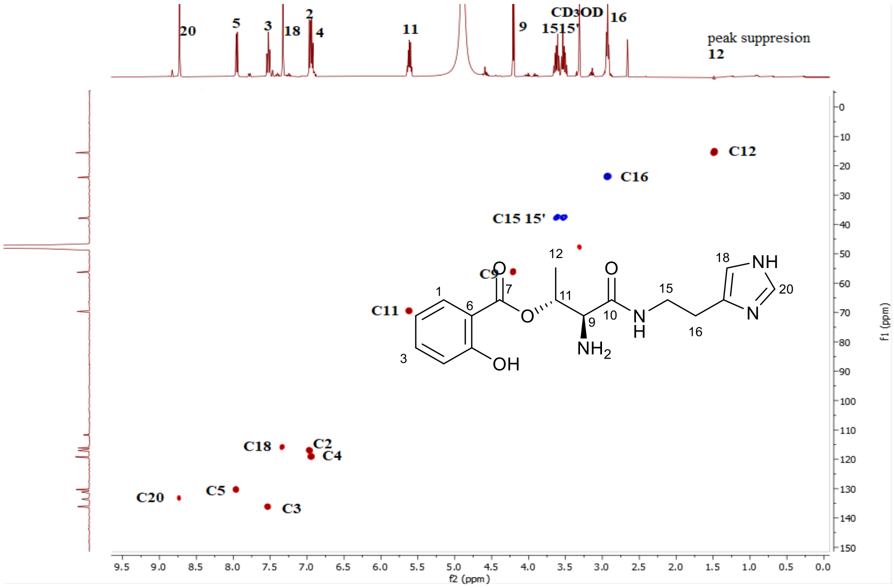

Figure S10. HSQC NMR spectrum of **2**

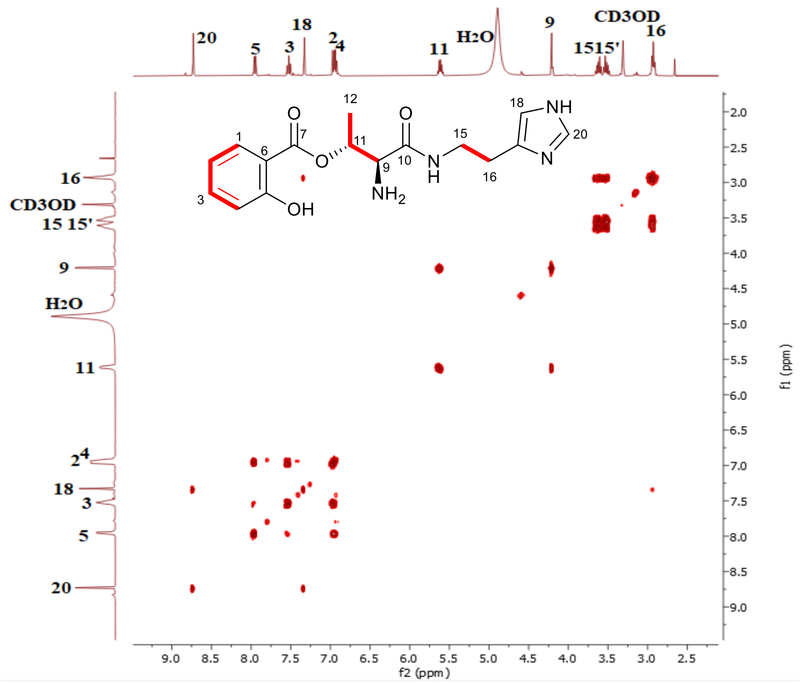

Figure S11. COSY NMR spectrum of **2**

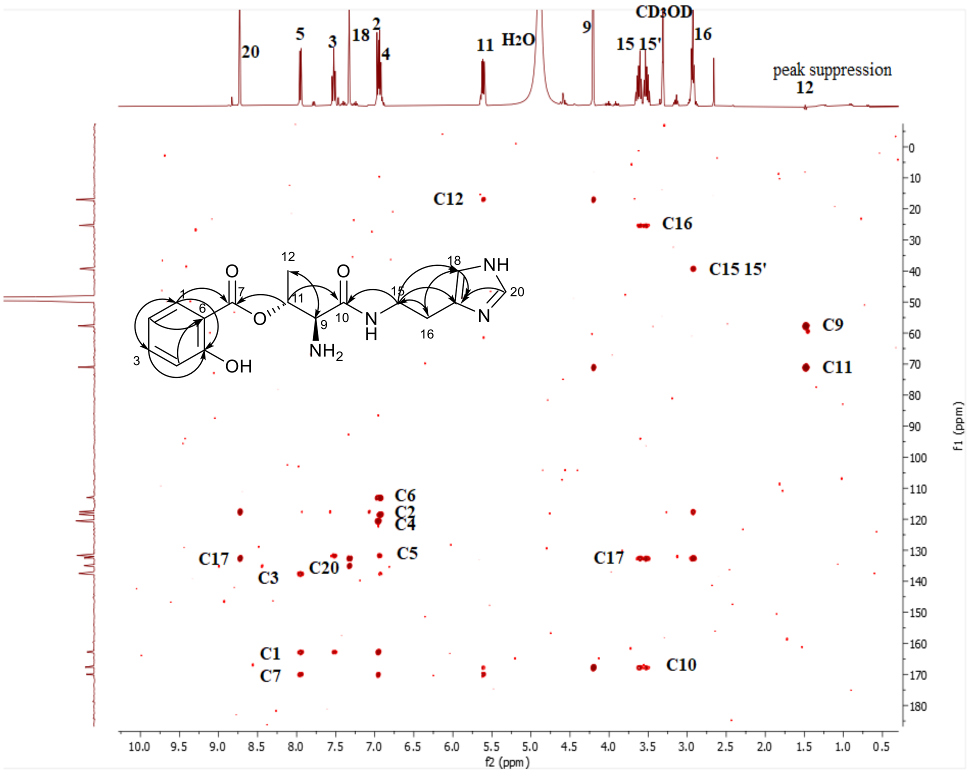

Figure S12. HMBC NMR spectrum of **2**

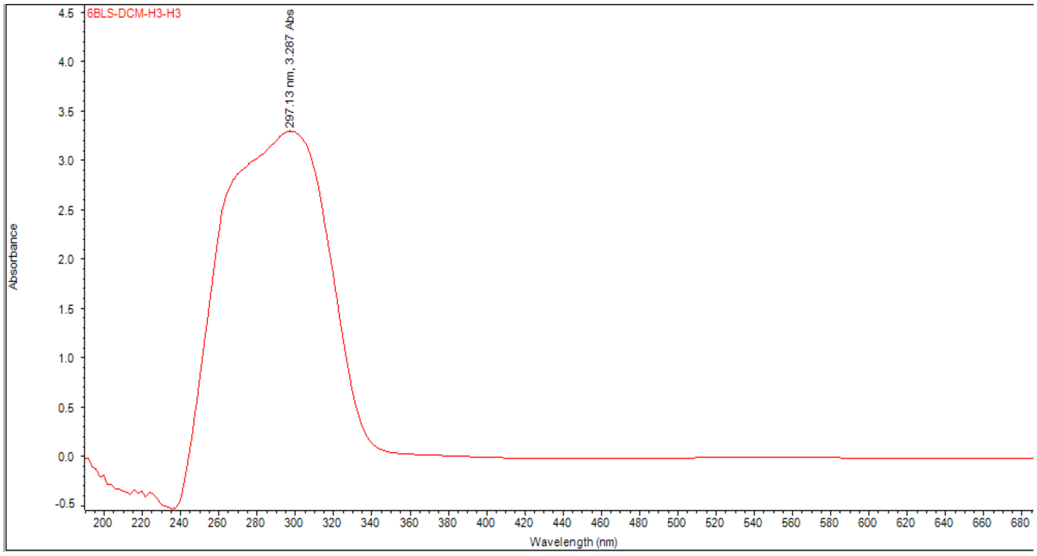

Figure S13. UV data of **2**

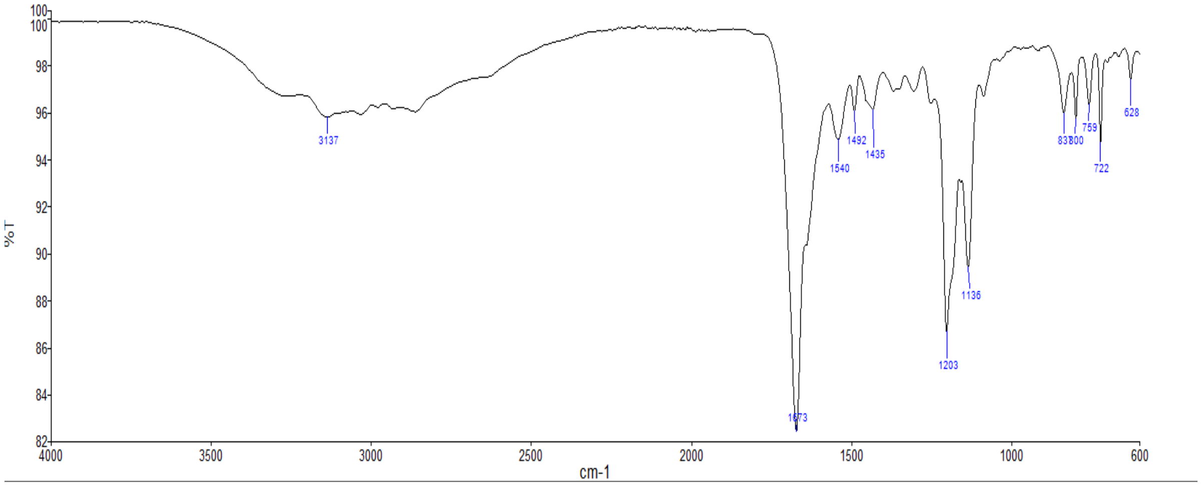

Figure S14. IR data of **2**

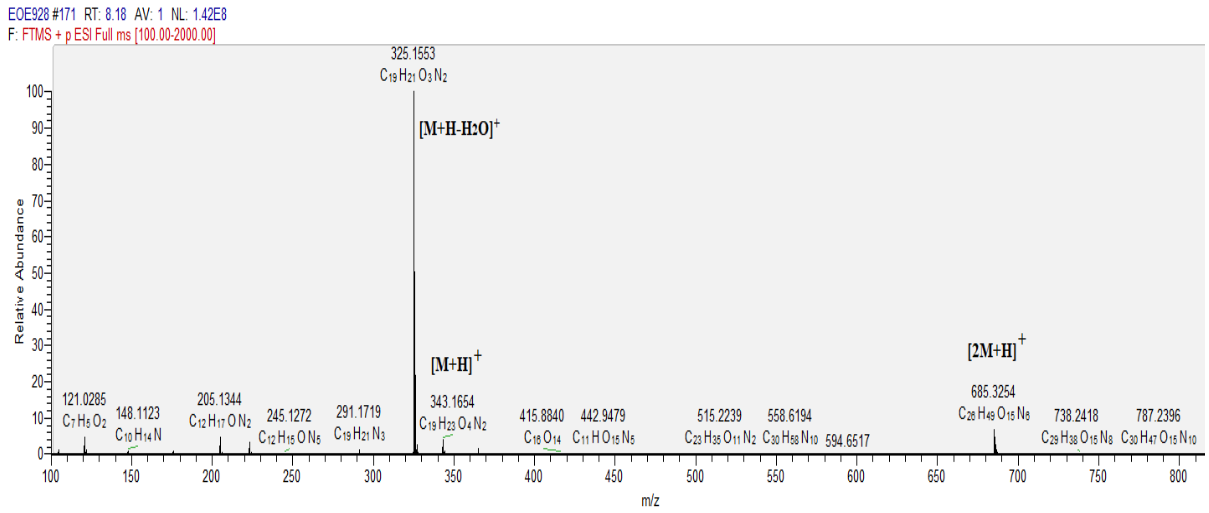

Figure S15. (+)-HR-ESIMS data of **3**

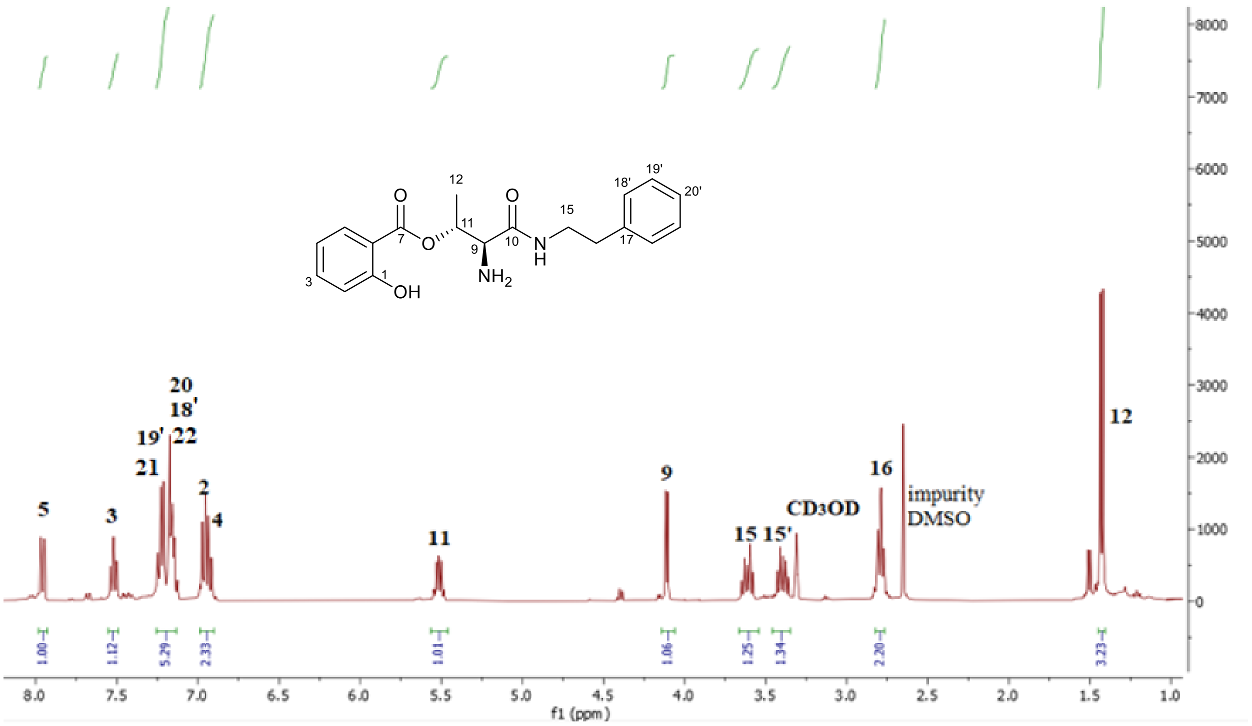

Figure S16. ^{1}H NMR spectrum of **3**

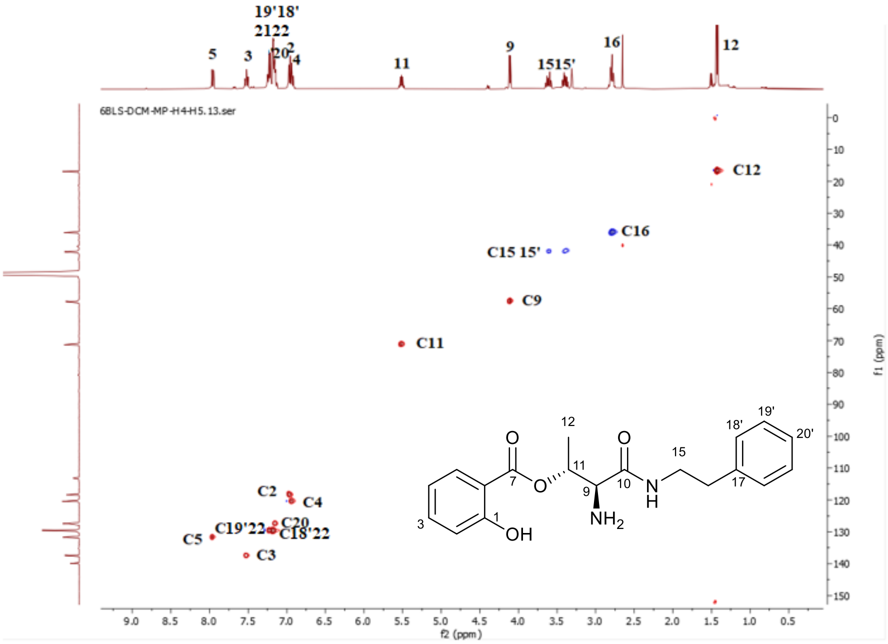

Figure S17. HSQC NMR spectrum **of 3**

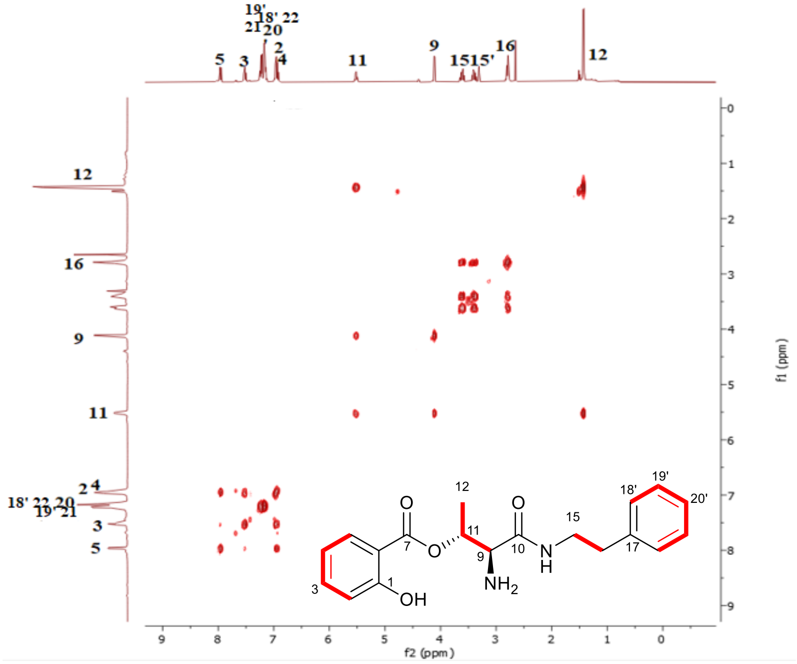

Figure S18. COSY NMR spectrum of **3**

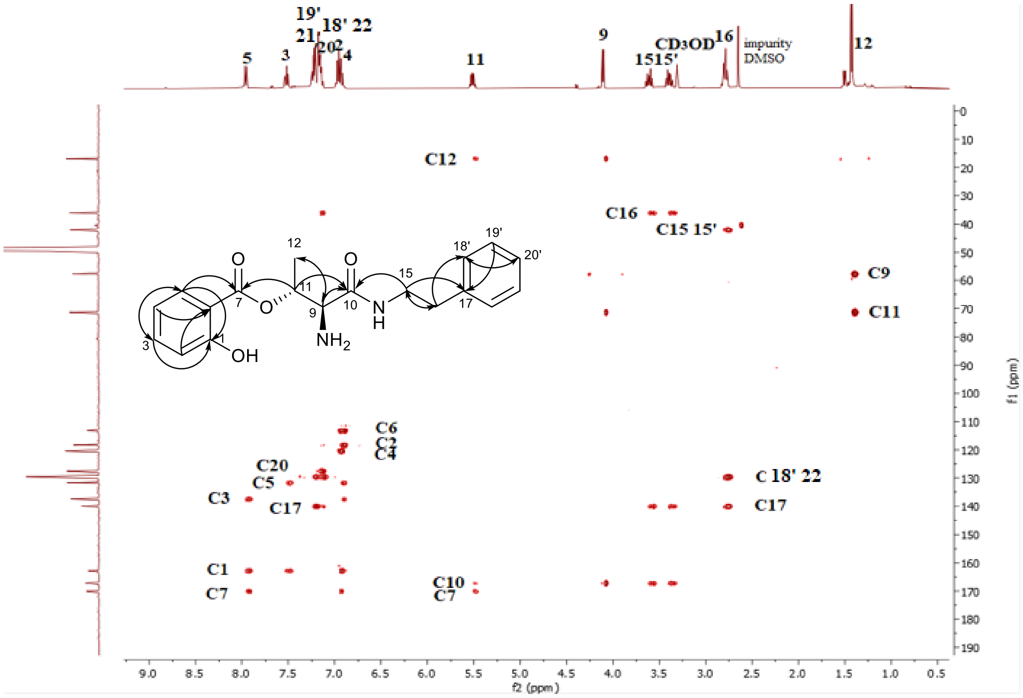

Figure S19. HMBC NMR spectrum of **3**

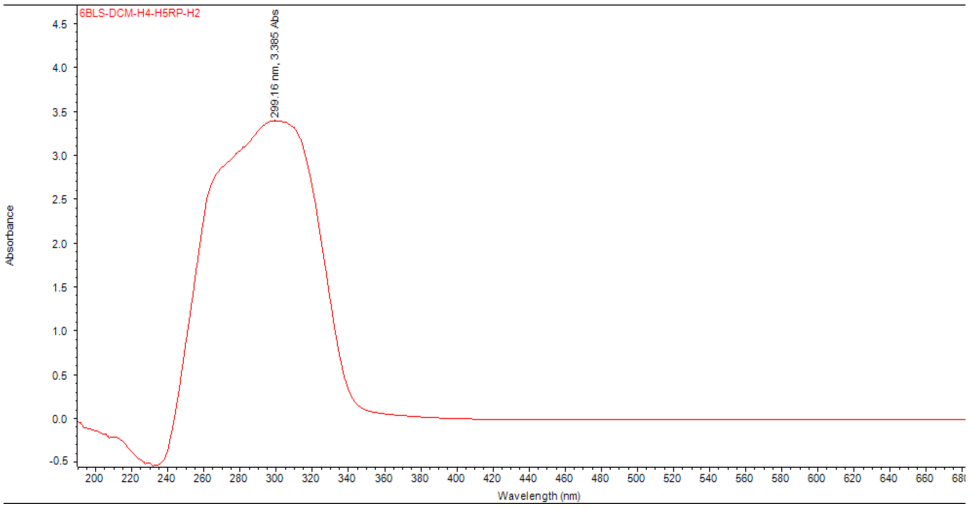

Figure S20. UV data of **3**

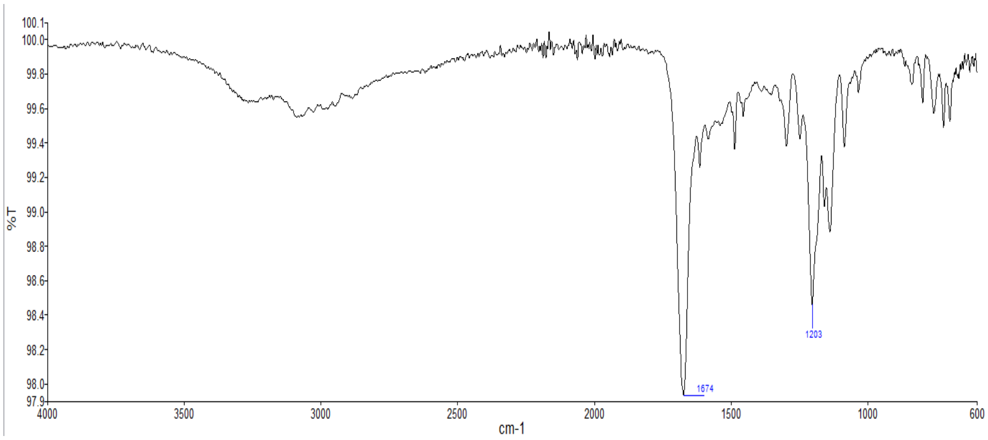

Figure S21. IR data of **3**

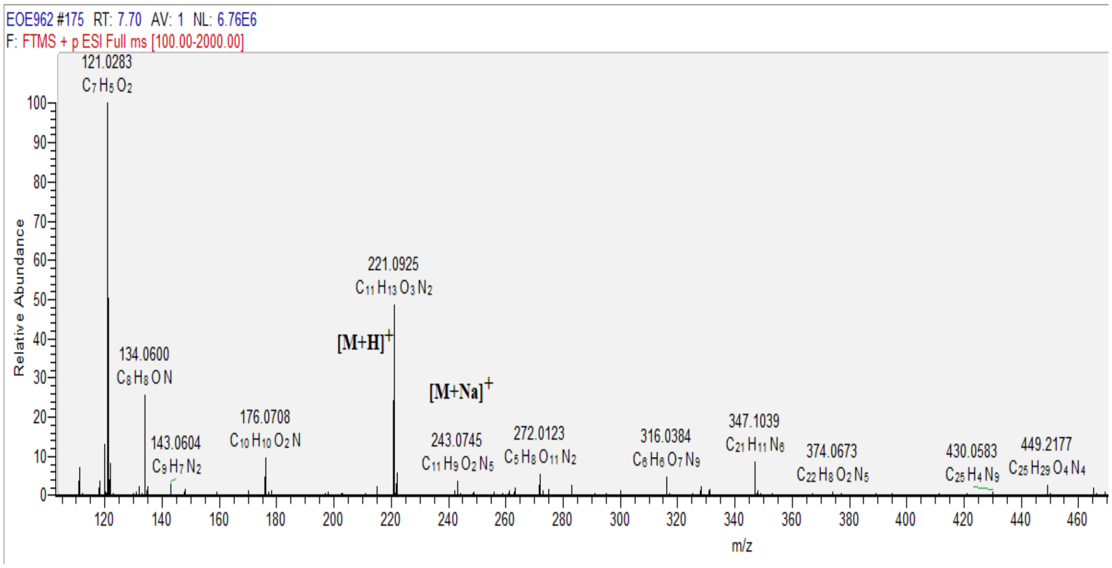

Figure S22. (+)-HR-ESIMS data of **4**

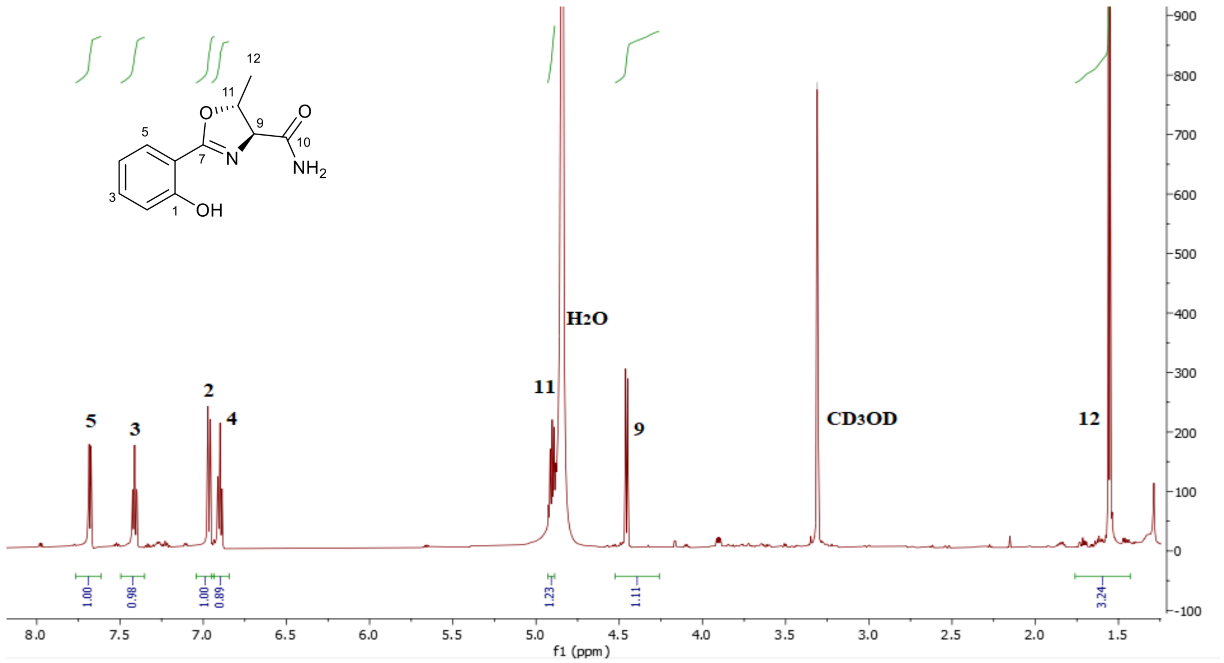

Figure S23. ^{1}H NMR spectrum of **4**

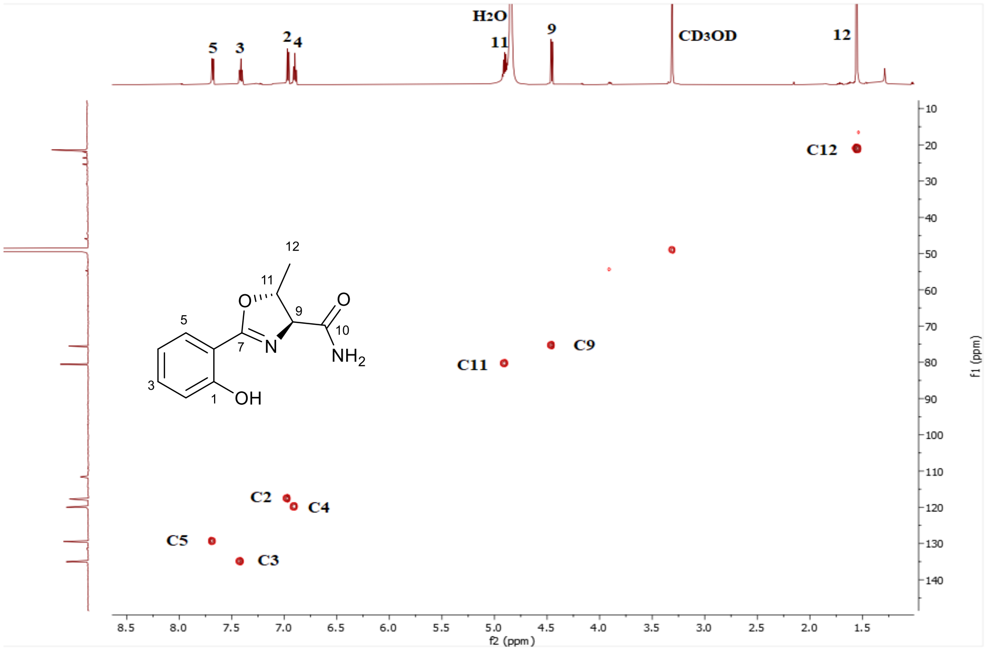

Figure S24. HSQC NMR spectrum of **4**

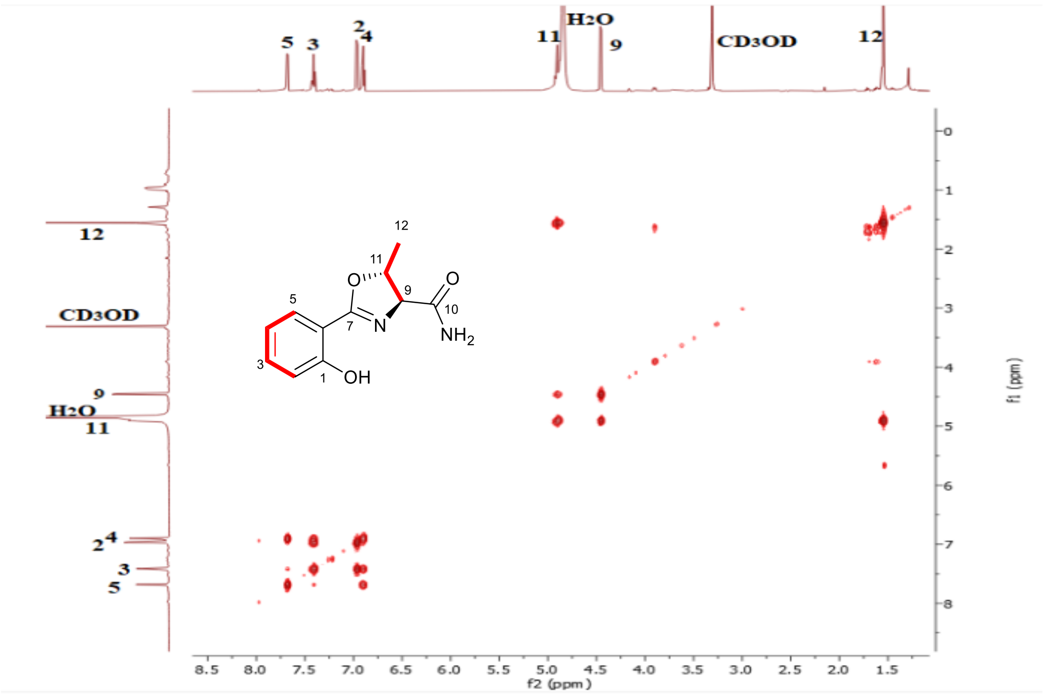

Figure S25. COSY NMR spectrum of **4**

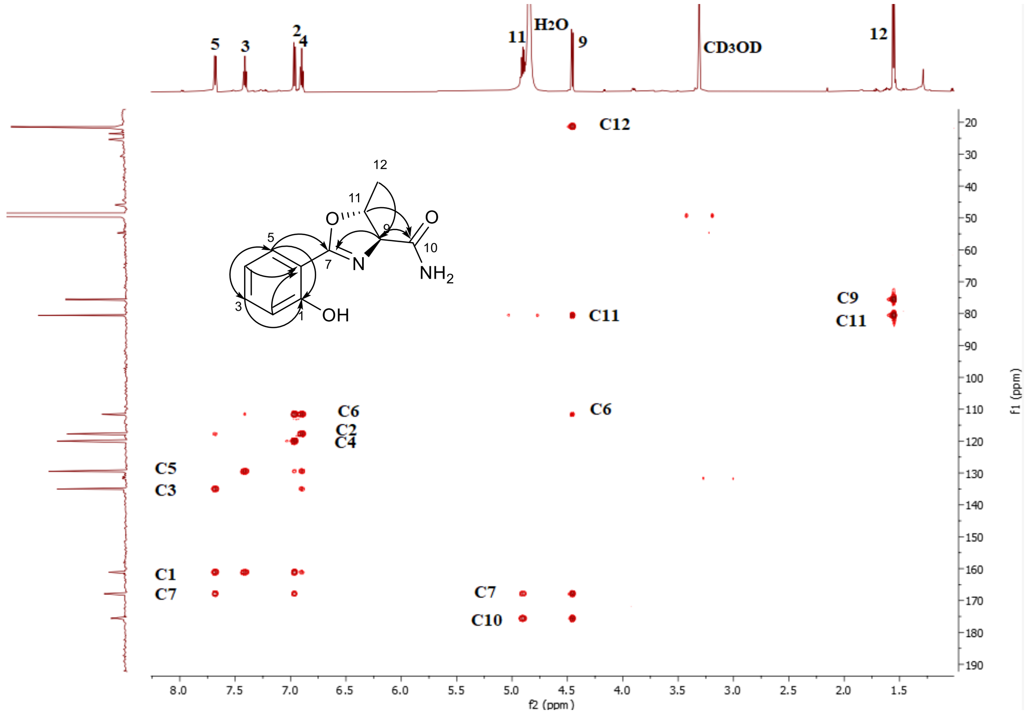

Figure S26. HMBC NMR spectrum of **4**

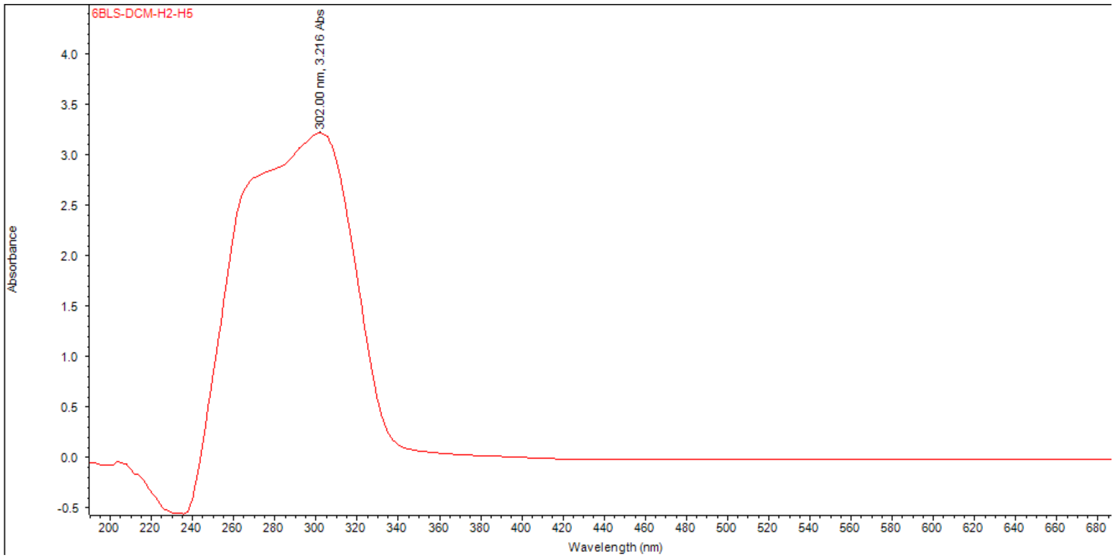

Figure S27. UV data of **4**

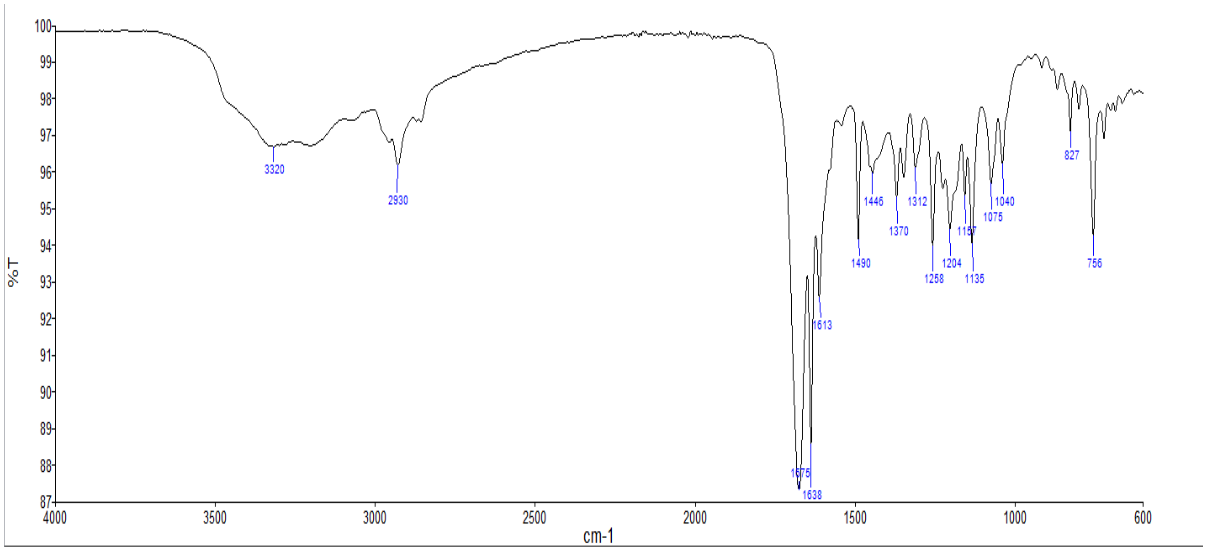

Figure S28. IR data of **4**

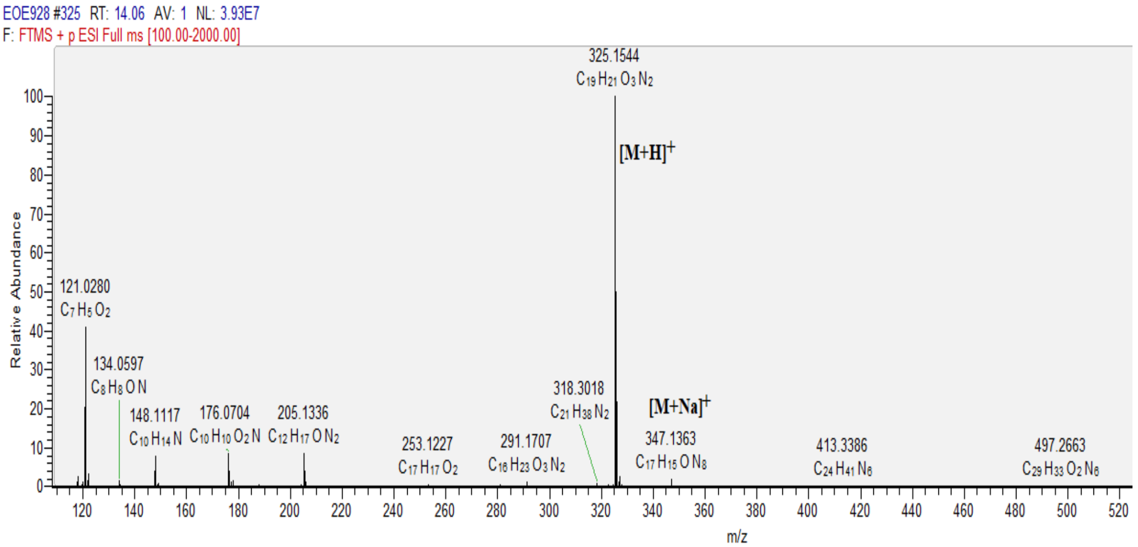

Figure S29. (+)-HR-ESIMS data of **5**

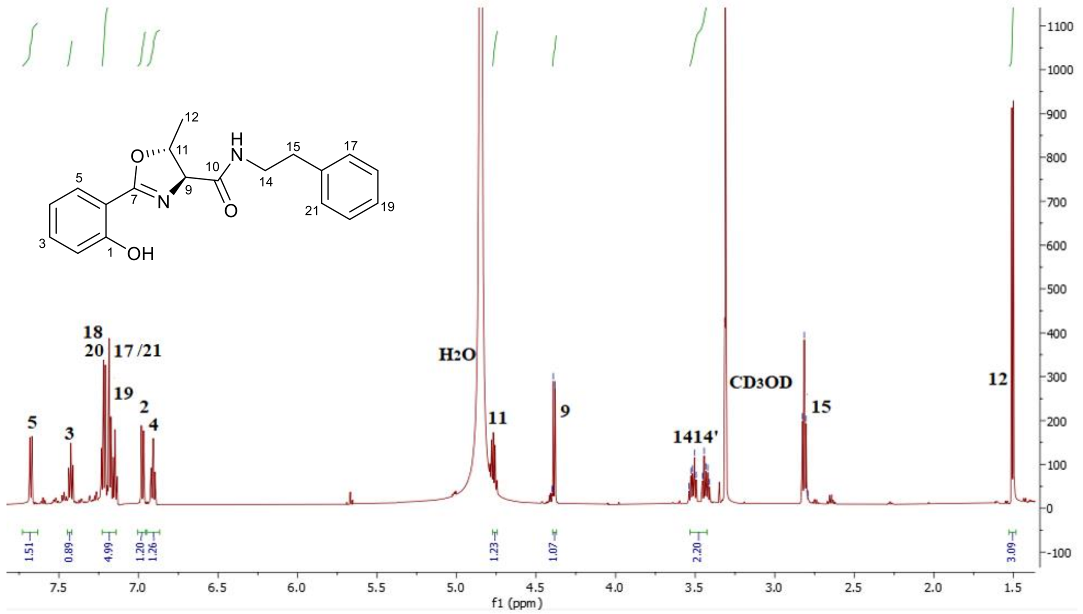

Figure S30. ^{1}H NMR spectrum of **5**

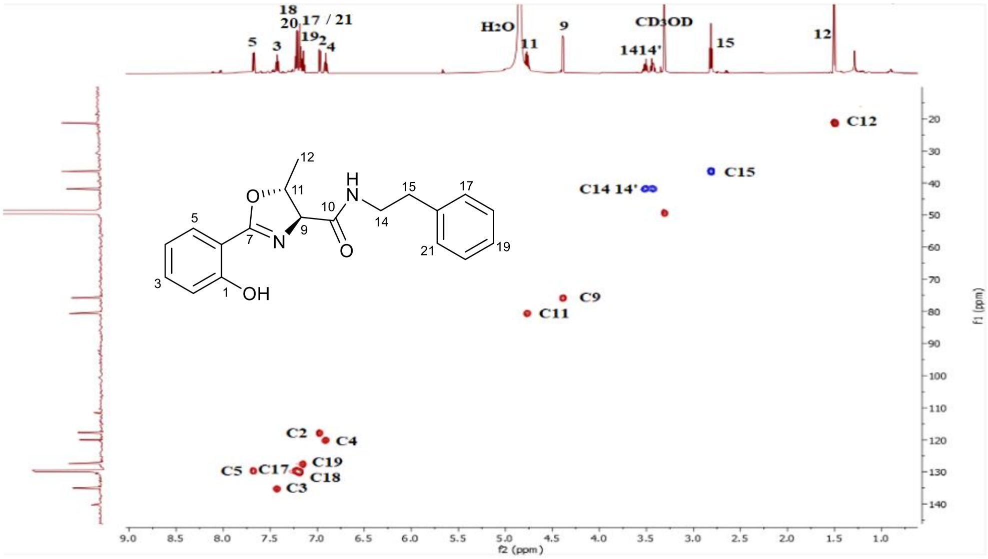

Figure S31. HSQC NMR spectrum of **5**

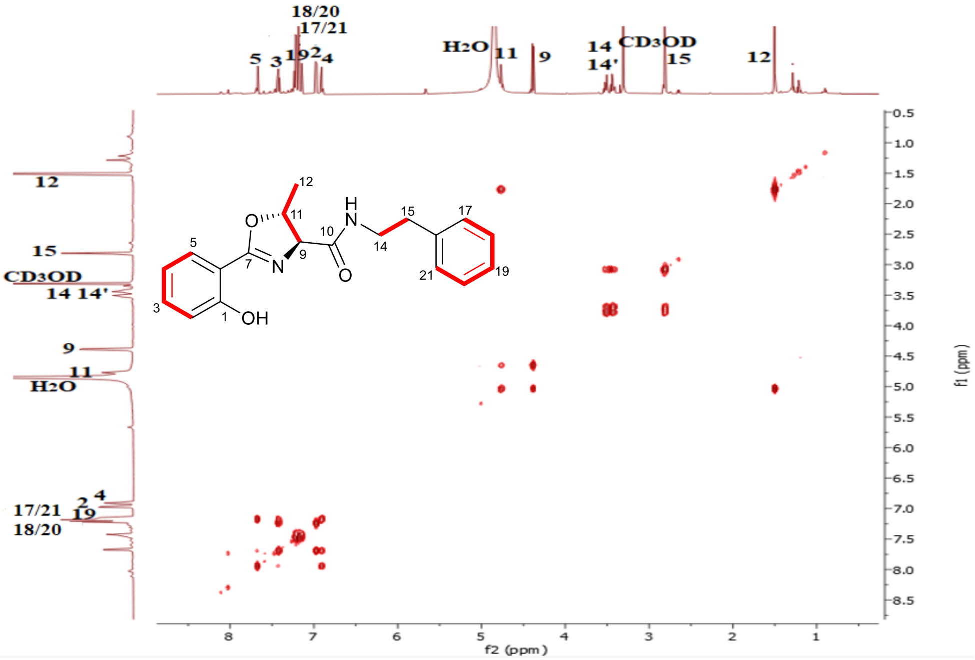

Figure S32. COSY NMR spectrum of **5**

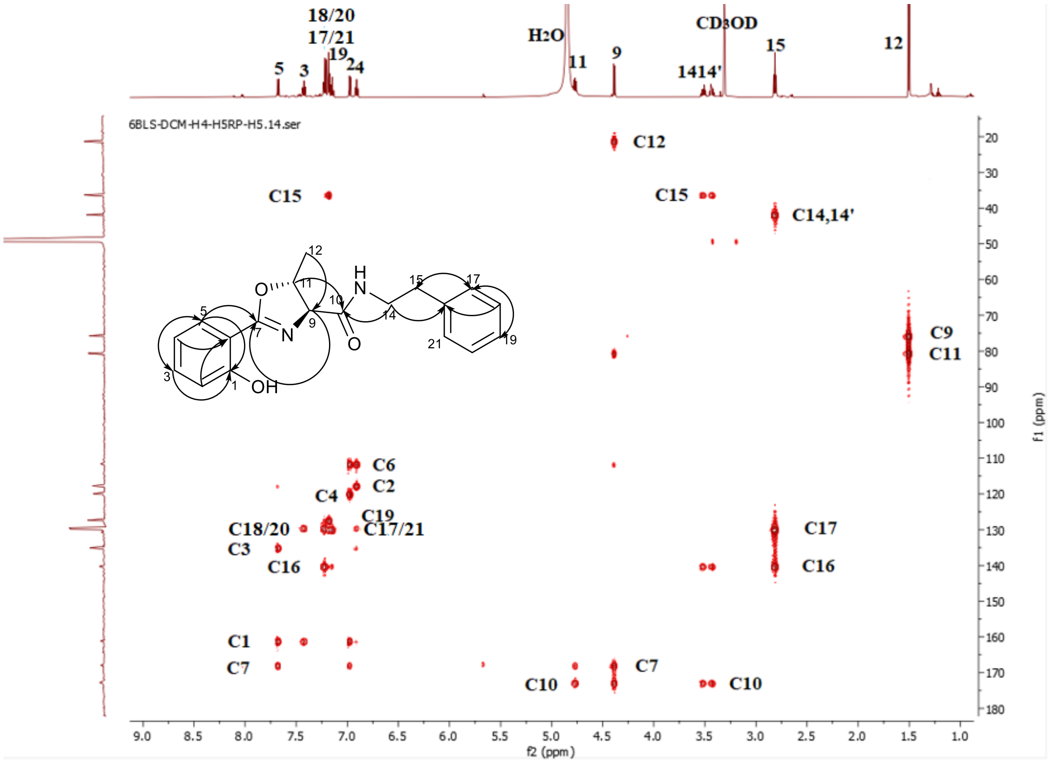

Figure S33. HMBC NMR spectrum of **5**

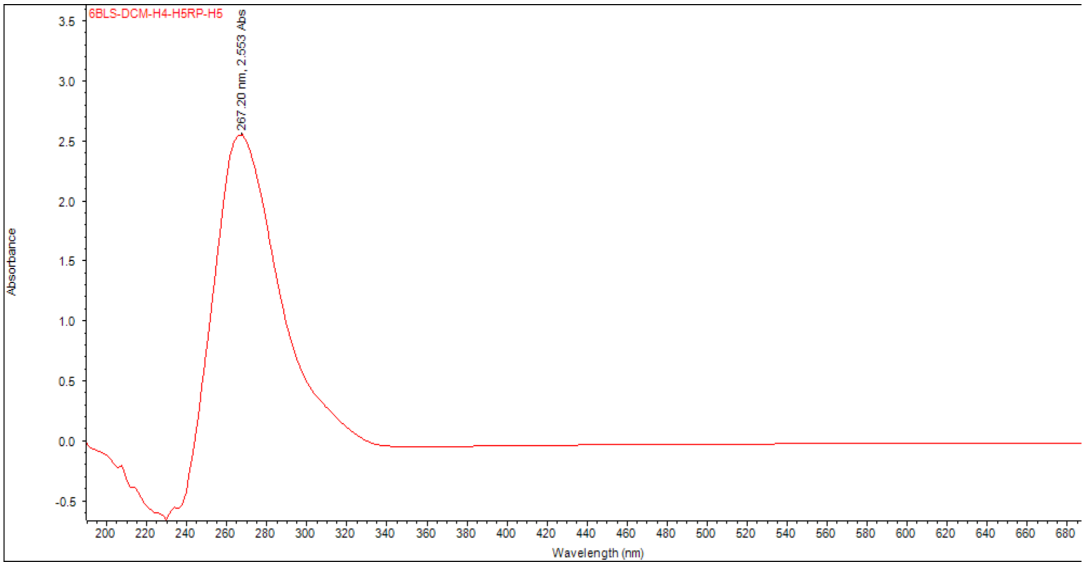

Figure S34. UV data of **5**

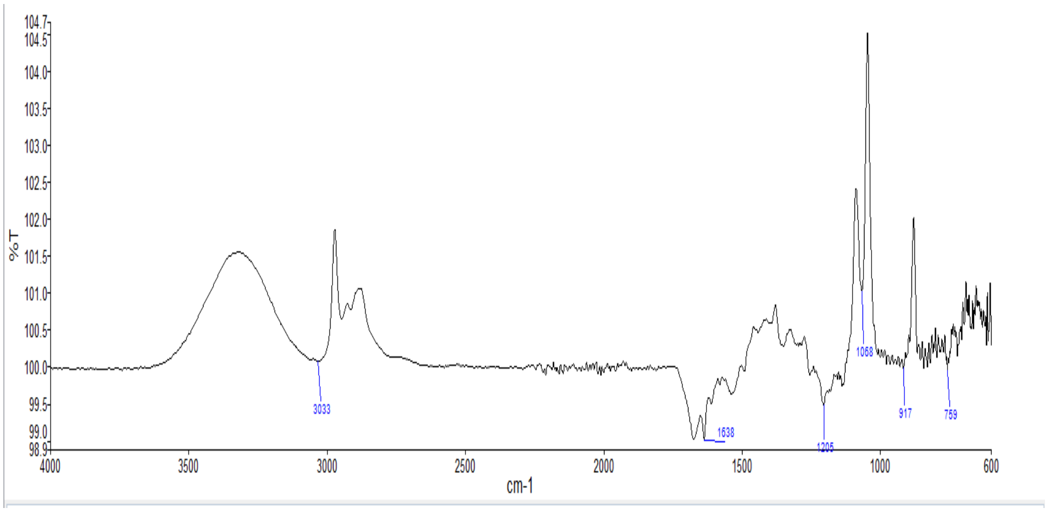

Figure S35. IR data of **5**

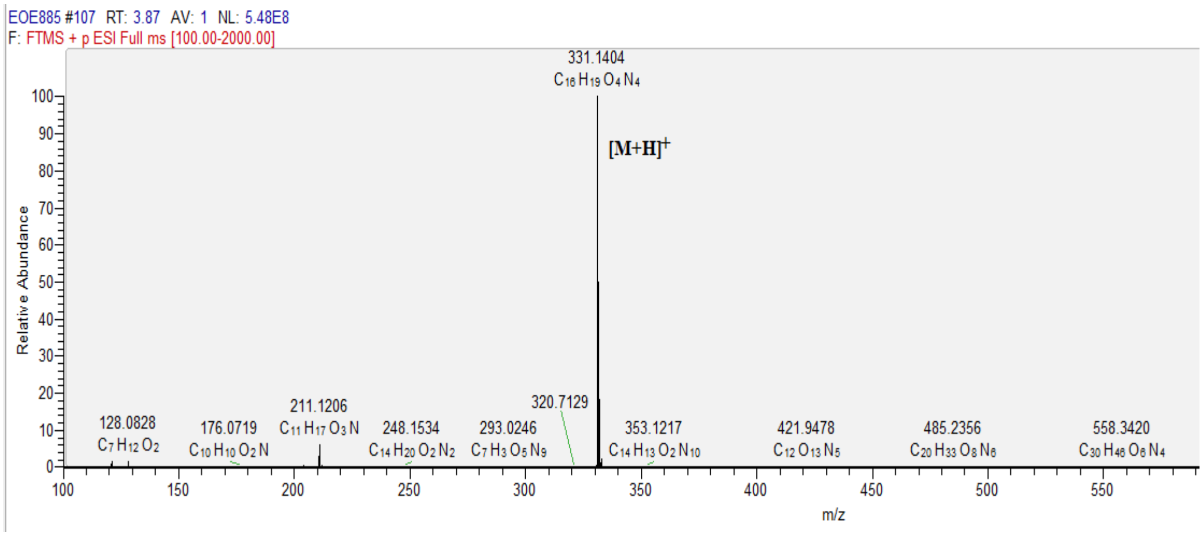

Figure S36. (+)-HR-ESIMS data of **6**

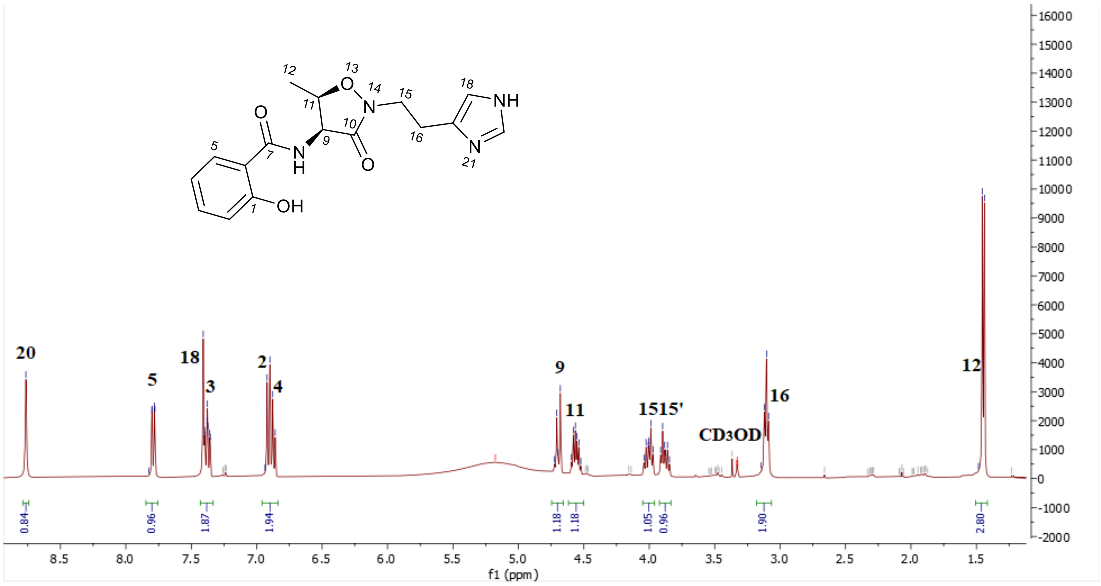

Figure S37. ^{1}H NMR spectrum of **6**

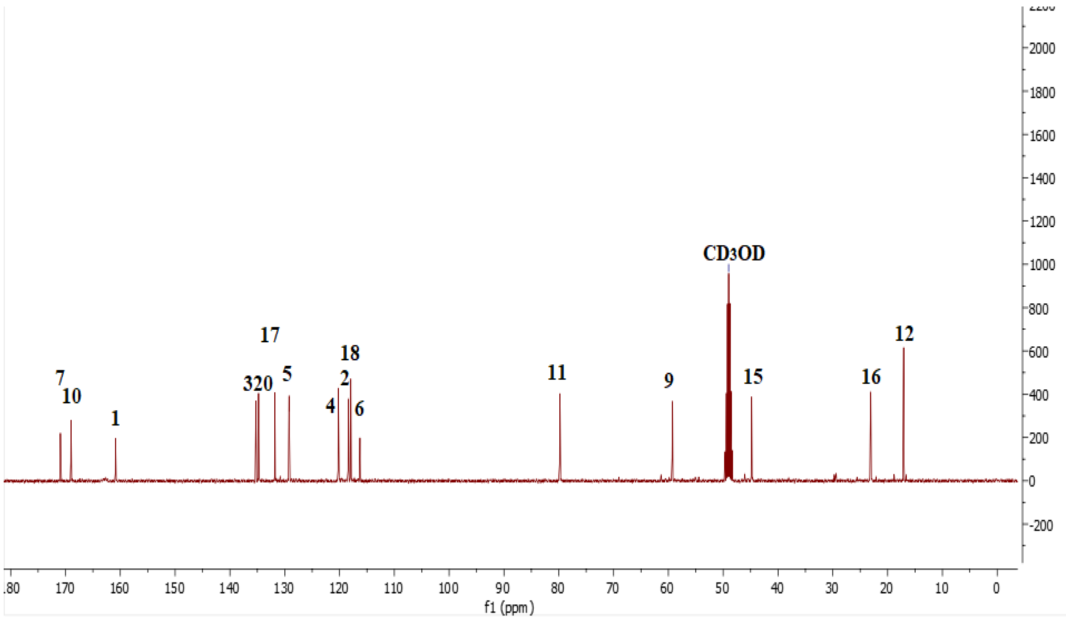

Figure S38. ^{13}C NMR spectrum of **6**

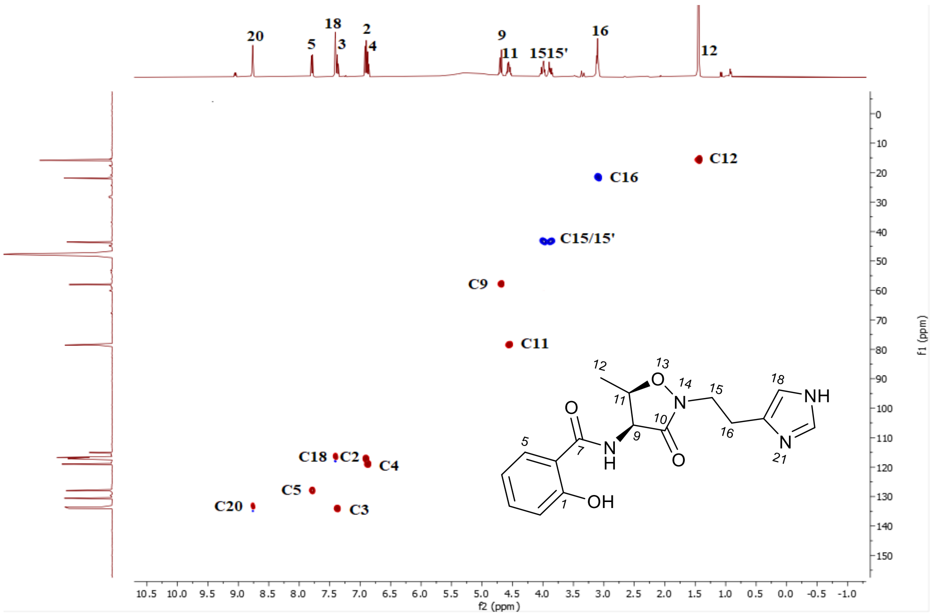

Figure S39. HSQC NMR spectrum of **6**

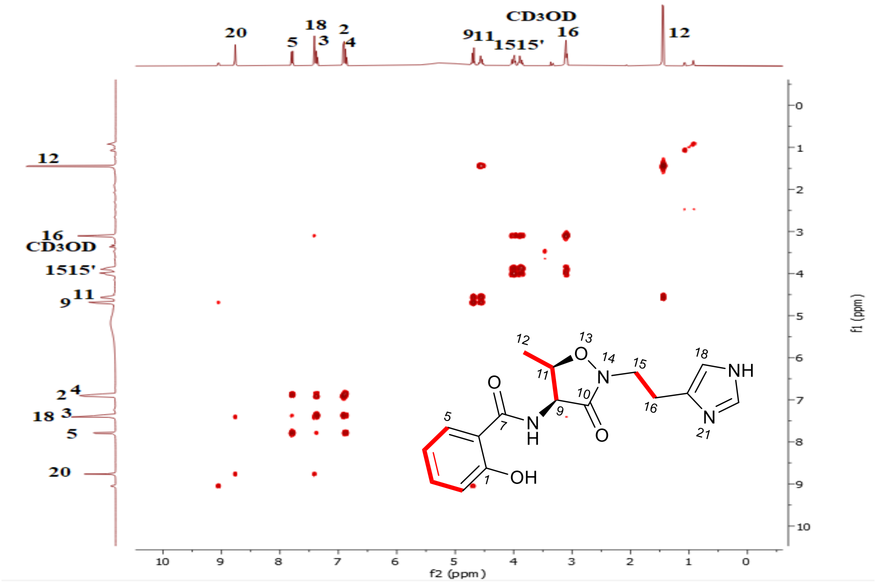

Figure S40. COSY NMR spectrum of **6**

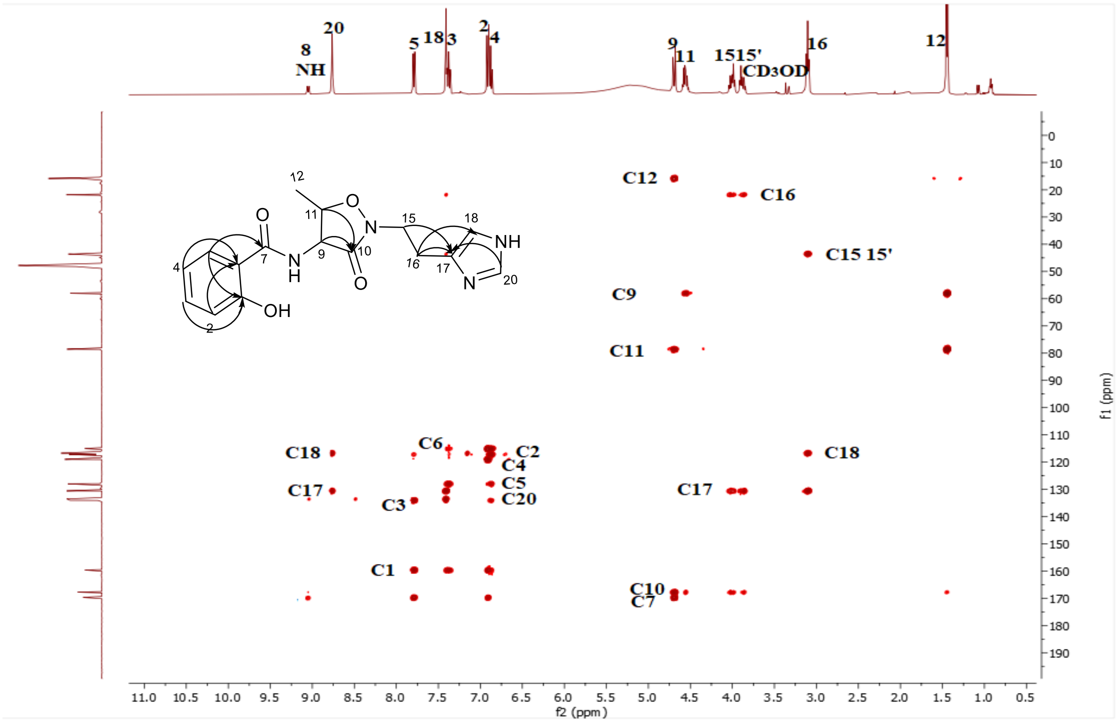

Figure S41. HMBC NMR spectrum of **6**

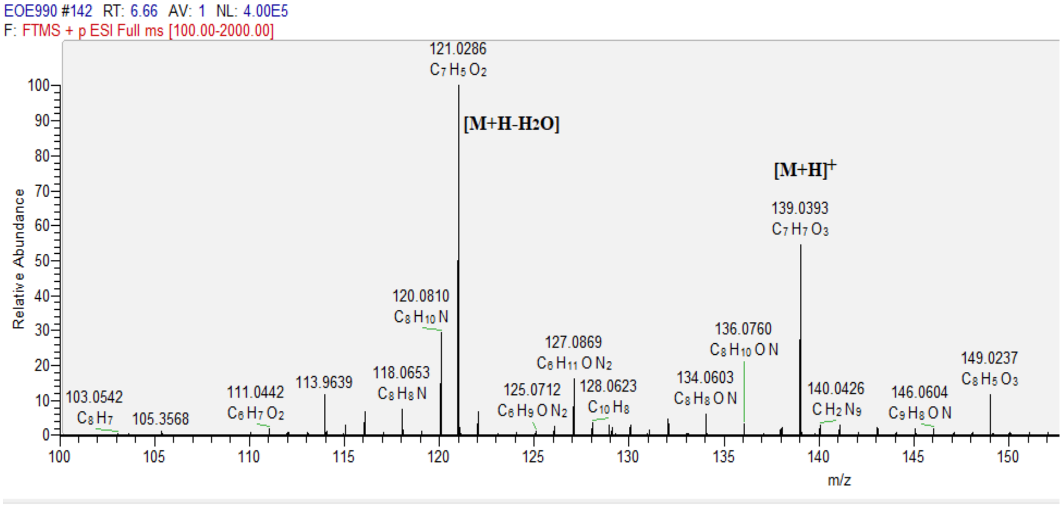

Figure S42. (+)-HR-ESIMS data of **7**

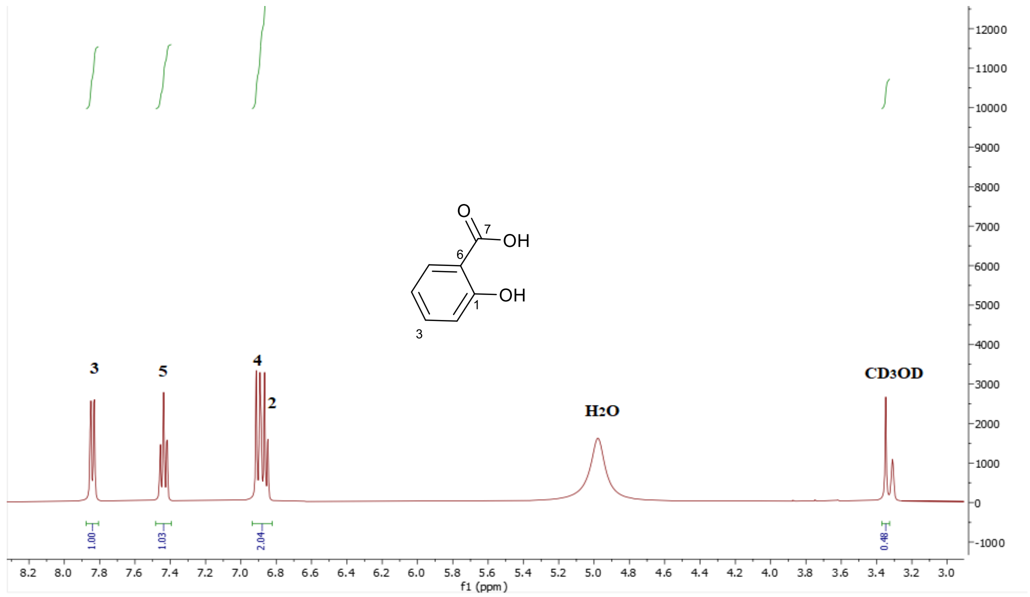

Figure S43. ^{1}H NMR spectrum of **7**

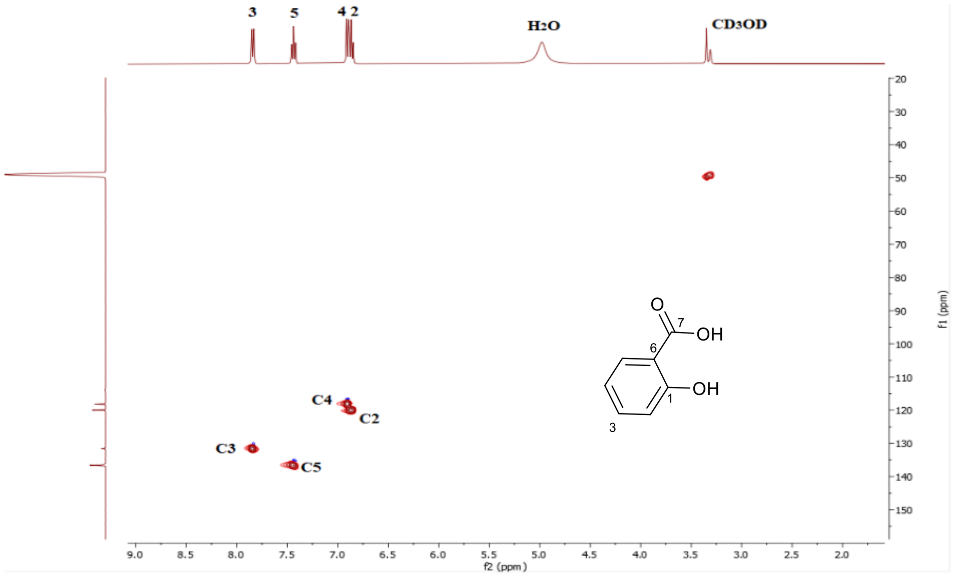

Figure S44. HSQC NMR spectrum of **7**

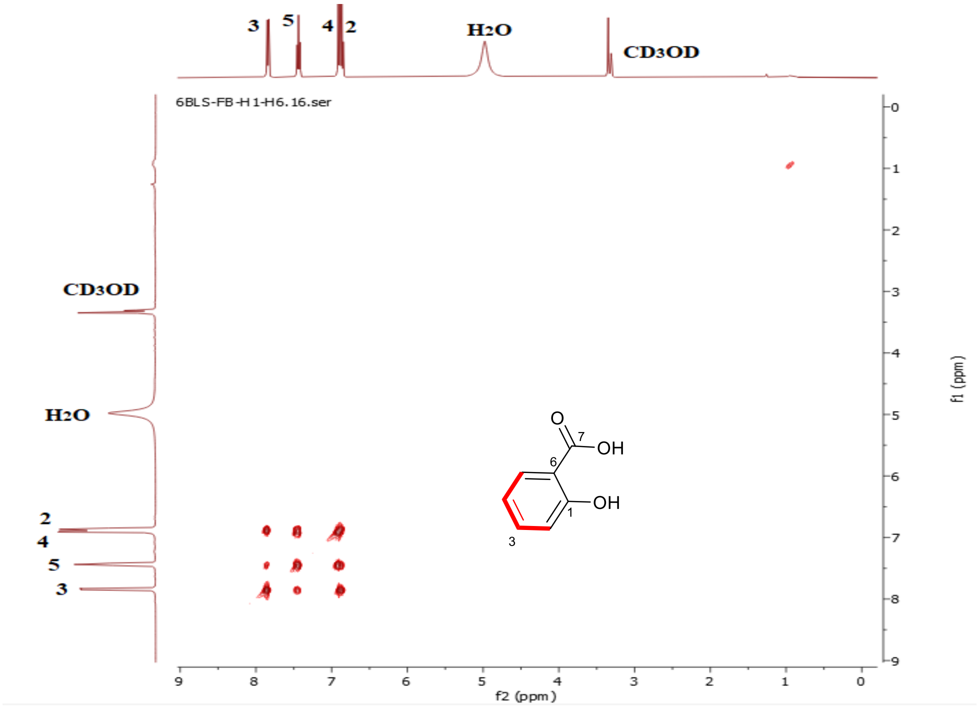

Figure S45. COSY NMR spectrum of **7**

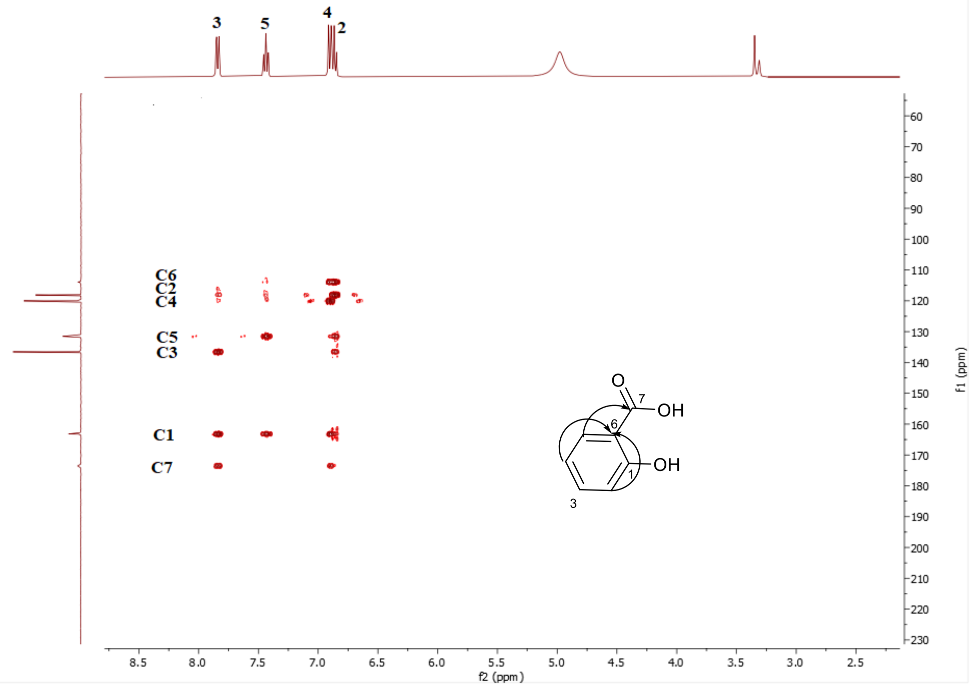

Figure S46. HMBC NMR spectrum of **7**

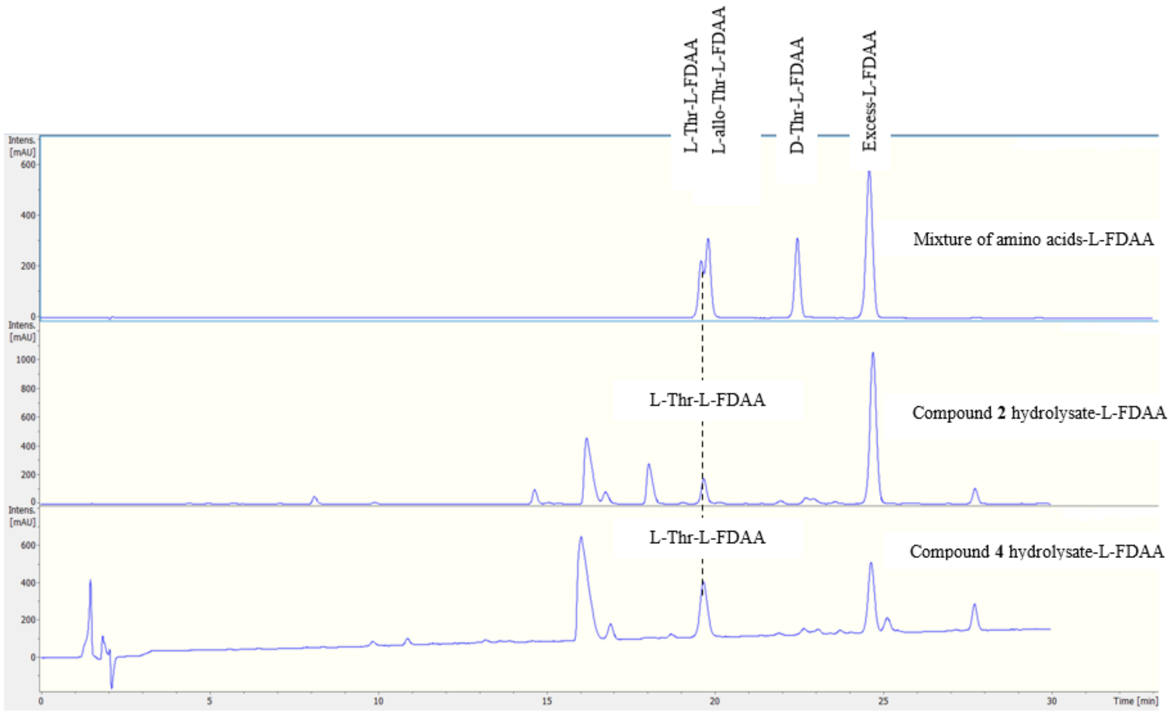

Figure S47 Chromatographic profiles of the compound (**2**) and (**4**) derivatized with L-FDAA and the standard amino acids with L-FDAA.

CCTANATCTTGAAAACAACTTCACCCCAGTCATGAATCACACCGTGGTAACCGTCCTCCCGA
AGGTTAGACTACCTACTTCTGGTGCAACCCACTCCCATGGTGTGACGGGCGGTGTGTACAAG
GCCCGGGAACGTATTCACCGCGACATTCTGATTCGCGATTACTAGCGATTCCGACTTCACGC
AGTCGAGTTGCAGACTGCGATCCGGACTACGATCGGTTTTATGGGATTAGCTCCACCTCGCG
GCTTGGCAACCCTCTGTACCGACCATTGTAGCACGTGTGTAGCCCAGGCCGTAAGGGCCATG
ATGACTTGACGTCATCCCCACCTTCCTCCGGTTTGTCACCGGCAGTCTCCTTAGAGTGCCCAC
CATAACGTGCTGGTAACTAAGGACAAGGGTTGCGCTCGTTACGGGACTTAACCCAACATCTC
ACGACACGAGCTGACGACAGCCATGCAGCACCTGTCTCAATGTTCCCGAAGGCACCAATCT
ATCTCTAGAAAGTTCATTGGATGTCAAGGCCTGGTAAGGTTCTTCGCGTTGCTTCGAATTAA
ACCACATGCTCCACCGCTTGTGCGGGCCCCCGTCAATTCATTTGAGTTTTAACCTTGCGGCCG
TACTCCCCAGGCGGTCAACTTAATGCGTTAGCTGCGCCACTAAAAGCTCAAGGCTTCCAACG
GCTAGTTGACATCGTTTACGGCGTGGACTACCAGGGTATCTAATCCTGTTTGCTCCCCACGCT
TTCGCACCTCAGTGTCAGTATTAGTCCAGGTGGTCGCCTTCGCCACTGGTGTTCCTTCCTATA
TCTACGCATTTCACCGCTACACAGGAAATTCCACCACCCTCTACCATACTCTAGTCAGTCAG
TTTTGAATGCAGCTCCCAGGTTGAACCCGGGGATTTCACATCCAACTTATCATACCACCTAC
GTGCGCTTTACGCCCATAATTCCGATCAACCCTTGCACCCTCTNATTTACCATG

Figure S48 DNA sequence of the bacterial strain UIAU-6B

A

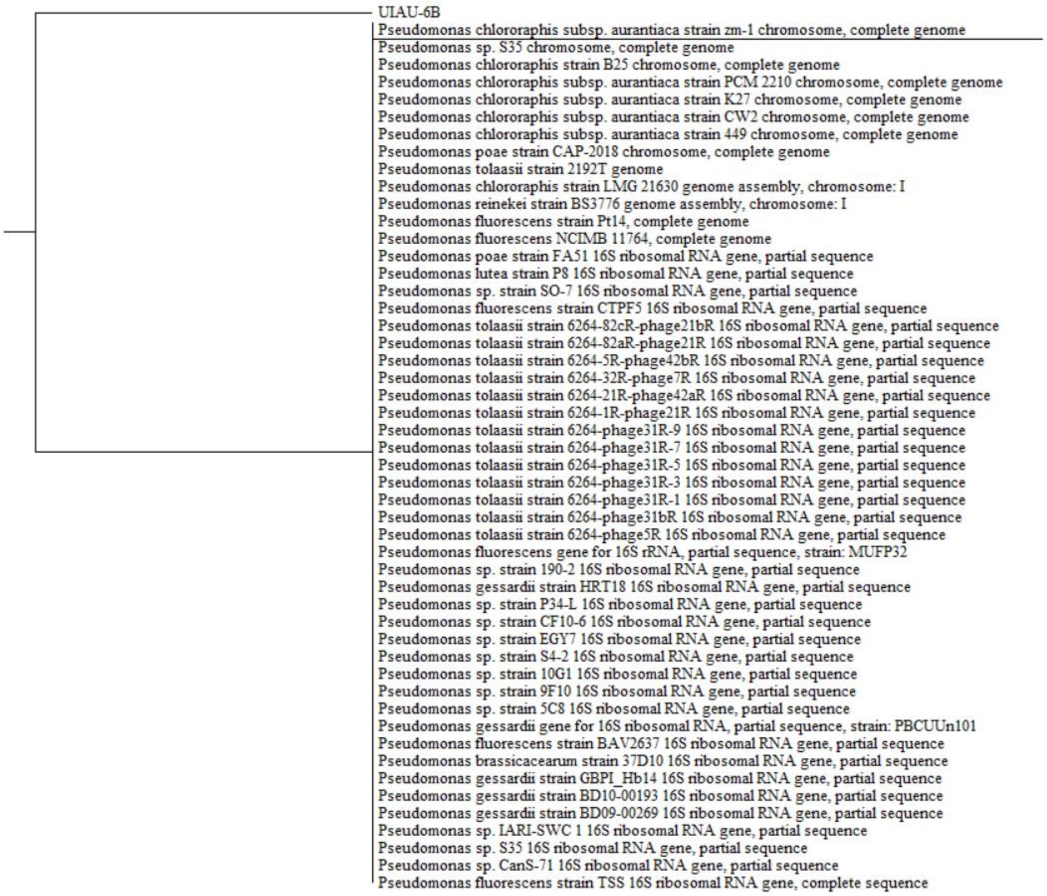

B

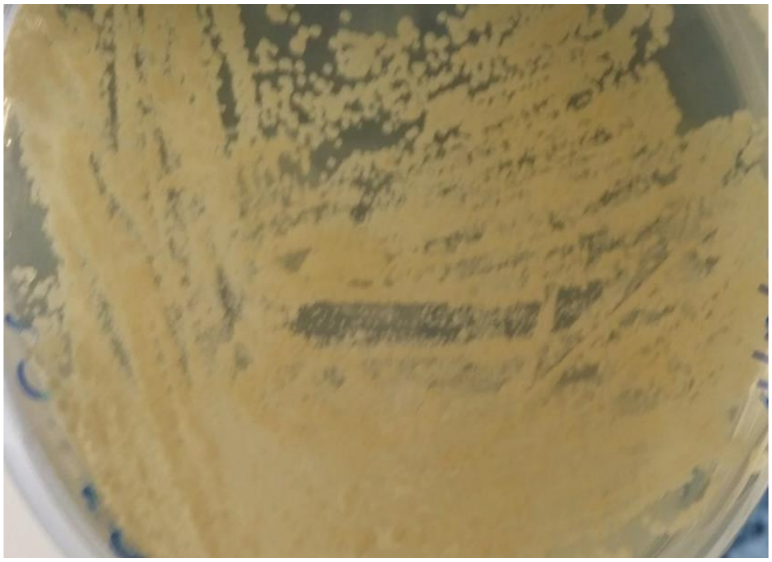

Figure S49 (A) Phylogenetic tree showing *Pseudomonad spp..* (**B**) Photo of *Pseudomonad spp* growing on ISP2 an agar plate

Table S1 NMR data for **1** (600 MHz, CD_3OD)

Position	δ_C, mult.	δ_H, mult. (*J* in Hz)	COSY	HMBC H → C
1	162.9, C			
2	118.2, CH	6.96, dd (8.7, 1.8)	3	1, 4, 5w, 6, 7
3	137.2, CH	7.52, td (8.7, 1.8)	2, 4	1, 2w, 4w, 5, 6w
4	120.2, CH	6.94, td (8.7, 1.8)	3, 5	1, 2, 3, 5, 6, 7w
5	131.6, CH	7.99, dd (8.7, 1.8)	4	1, 2w, 3, 7
6	113.2, C			
7	170.0, C			
9	57.6, CH	4.20, d (6.5)	11	10, 11, 12
10	169.4, C			
11	71.1, CH	5.67, qd (6.6, 6.5)	9, 12	7, 10, 12
12	16.7, CH_3	1.54, d (6.6)	9	9, 10

w= weak HMBC intensity.

Table S2 NMR data for **2** (600 MHz, CD_3OD)

Position	δ_C, mult.	δ_H, mult. (*J* in Hz)	COSY	HMBC H → C
1	162.8, C			
2	118.4, CH	6.96, d (8.7)	3	1, 4, 7
3	137.5, CH	7.52, td (8.7, 1.7)	2, 4	1, 2, 5, 6, 7
4	120.6, CH	6.94, dd (8.7, 1.7)	3, 5	1, 2
5	131.7, CH	7.95, dd (8.7, 1.7)	4	3, 6
6	113.1, C			
7	169.9, C			
9	57.7, CH	4.21, d (6.5)	11	11, 12, 10
10	167.7, C			
11	71.0, CH	5.61, qd (6.6, 6.5)	9, 12	7, 10, 12
12	17.0, CH_3	1.49, d (6.1)	11	9, 11
15,15’	39.3, CH_2	3.62, dt (13.8, 6.8); 3.52, dt (13.8, 6.8)	16	10, 16, 17
16	25.3, CH_2	2.92, m	15	15, 17, 18
17	132.5, C			
18	117.5 CH	7.33, d (0.6)	20	17, 20
20	135.0, CH	8.73, d 0.6)	18	17, 18

Table S3 NMR data for **3** (600 MHz, CD_3OD)

Position	δ_C, mult.	δ_H, mult. (*J* in Hz)	COSY	HMBC H⟶C
1	162.8, C			
2	118.4, CH	6.98, m	3	1, 4, 6, 7
3	137.4, CH	7.54, td (8.7, 1.8)	2, 4	1, 5
4	120.5, CH	6.95, m	3, 5	2, 3, 5, 6
5	131.7, CH	7.98, dd (8.7, 1.8)	4	1, 3, 7
6	113.2, C			
7	170.0, C			
9	57.8, CH	4.13, d (6.5)	11	10, 11, 12
10	167.2, C			
11	71.3, CH	5.53, dq (6.5, 6.5)	9, 12	7,10, 12
12	16.9, CH_3	1.43, d (6.5)	11	9, 11
15	42.1, CH_2	3.44, dt (13.7, 7.2)<br>3.64, dt (13.7, 7.2)	16	10, 16, 17
16	36.1, CH_2	2.79, m	15, 15'	15, 17, 18', 22
17	140.0, C			
18', 22	129.5, CH	7.16 (m)	19', 21	17, 20', 22
19', 21	129.6, CH	7.22(m)	18', 22, 20'	17, 21
20'	127.5, CH	7.14 (m)	19, 21	17, 18', 22

Table S4 NMR data for **4** (600 MHz, CD_3OD)

Position	δ_C, mult.	δ_H, mult. (*J* in Hz)	COSY	HMBC ⟶ H C
1	161.1, C			
2	117.7, CH	6.96, d (8.3)	3	1, 4, 5, 6, 7
3	135.0, CH	7.41, ddd (8.3, 1.7)	2, 4	1, 2, 4w, 5, 6
4	119.9, CH	6.90, td (7.4, 1.7)	3, 5	1, 2, 3, 5, 6, 7
5	129.5, CH	7.68, dd(7.4, 1.7)	4	1, 2, 3, 7
6	111.6, C			
7	167.8, C			
9	75.5, CH	4.46, d (7.3)	11	5w, 6, 7, 10, 11, 12,
10	175.6, C			
11	80.6, CH	4.90, qd (6.3, 7.3)	9, 12	7, 9, 10
12	21.4, CH_3	1.57, d (6.3)	11	9, 11

w= weak HMBC intensity.

Table S5 NMR data for **5** (600 MHz, CD_3OD)

Position	δ_C, mult.	δ_H, mult. (*J* in Hz)	COSY	HMBC H ⟶ C
1	161.1, C			
2	117.7, CH	6.99, d (8.3)	3	1, 4, 6, 7
3	135.1, CH	7.43, td (8.3, 1.7)	2, 4	1, 5, 6
4	120.0, CH	6.92, t (7.4, 1.7)	3, 5	2, 3, 5, 6
5	129.5, CH	7.67, dd (7.4, 1.7)	4	1, 2, 3, 7
6	111.6, C			
7	168.0, C			
9	75.8, CH	4.38, d (7.3)	11	6, 7, 10, 11, 12
10	172.9, C			
11	80.6, CH	4.78, dq (6.3, 7.3)	9, 12	7, 10
12	21.3, CH_3	1.51, d (6.3)	11	9, 11
14	41.9, CH_2	3.44, dt (13.6, 7.2), 3.51, dt (13.6, 7.2)	15	10, 15, 16
15	36.3, CH_2	2.82, t (7.2)	14	14, 16, 17,21
16	140.2, C			
17/21	129.9, CH	7.20 (m)	18, 20	15, 21, 19
18/20	129.5, CH	7.22 (m)	17,21,19	16, 20
19	127.3, CH	7.15 (m)	18,21	16, 17/21

Table S6 NMR data for **6** (600 MHz, CD_3OD)

Position	δ_C, mult.	δ_C [19]	δ_H, mult. (*J* in Hz)	HMBC H ⟶ C
1	160.9, C	161.1		
2	118.4, CH	118.5	6.88, t (8.0)	1,4, 7, 6
3	135.3, CH	135.3	7.36, td (8.0, 1.6)	1, 5, 6
4	120.2, CH	120.3	6.89, dd (8.0, 1.6)	1w, 2, 3, 5, 6
5	129.2, CH	129.2	7.77, dd (8.0, 1.6)	1, 2w, 3, 7
6	116.3, C	116.5		
7	171.1, C	171.1		
9	59.25, CH	59.5	4.68, d (10.8)	7, 10, 11, 12
10	168.6, C	169.1		
11	79.9, CH	79.9	4.54, dq (10.8, 6.1)	9, 10
12	17.2, CH_3	17.2	1.45, d (6.1)	9, 11
15 15’	44.8, CH_2	45.1	3.99, dt (14.6, 6.5)<br>3.86, dt (14.6, 6.5)	10, 16, 17
16	23.1, CH_2	23.6	3.09, t (6.5)	15, 17,18
17	131.8, C	132.6		
18	117.9, CH	118.2	7.39, s	17, 20
20	134.8, CH	135.3	8.75, s	17,18

[19] Literature reference for **6** ^{13}C NMR. NMR solvents used for reported 6 in the literature was CD_3OD at 600 MHz w= weak HMBC intensity.

Table S7 NMR data for **7** (600 MHz, CD_3OD)

Position	δ_C, mult.	δ_C [30]	δ_H mult. (J in Hz)
1	163.2, C	161.7	
2	118.1, CH	116.8	6.91, d (8.3)
3	136.6, CH	135.5	7.43, td (8.3, 7.1)
4	120.0, CH	118.7	6.86, m
5	131.5, CH	129.9	7.83, dd (7.4, 1.7)
6	113.9, C	111.9	
7	173.5, C	171.4	

[30] Literature reference for **7** ^{13}C NMR. NMR solvents used for the reported in the literature was in CD_3OD at 300 MHz

Experimental procedures

Antibacterial assays

For the antibacterial tests, thawed stock inocula suspensions from cryovials of each microorganism are streaked onto MHII agar plates (Muller Hinton II) and incubated at 37 °C overnight to obtain isolated colonies. Single colonies of each microorganism are inoculated in 10 mL of MHII broth and incubated overnight at 37 °C with shaking at 220 rpm and then diluted in order to obtain assay inocula of approximately 5–6 × 10^5 CFU/mL.

For the assay 90 µL/well of the diluted inoculum are mixed with 1.6 µL/well of each compound (**1**–**7**) dissolved in DMSO and 8.4 µL/well of MHII medium. Absorbance at 612 nm is measured with a ENVISION, Perkin Elmer™spectrophotometer at T_0 (zero time) and immediately after that, plates are statically incubated at 37 °C for 20 h. After this period, the assay plates are shaken using the DPC Micromix-5 and once more the absorbance at OD 612 nm is measured at T_f (final time). Percentage inhibition of growth is calculated using the following normalization:

% Inhibition = [1 − [(T_fSample − T_0Sample) − (T_fBlank − T_0blank)/(T_fGrowth − T_0Growth) − (T_fBlank − T_0blank)] × 100.

Each compound is serially diluted in DMSO with a dilution factor of 2 to provide 10 concentrations starting at 64 µg/mL for all the assays. The MIC is defined as the lowest concentration of an antimicrobial or antifungal compound that inhibited ≥ 90% of the growth of a microorganism after overnight incubation. The data are analyzed using the Genedata Screener program (Genedata AG, Switzerland). In all experiments performed in this work the RZ' factor obtained was between 0.85 and 0.95.

Antimycobacterium Tuberculosis assay

The minimum inhibitory concentration (MIC) values were determined using the previously reported standard broth microdilution method [1]. A 10 mL culture of *M. tuberculosis* H37Rv [2] was grown to an absorbance (OD600) of 0.6–0.7 in Middlebrook 7H9 medium (Difco) supplemented with 0.2% glucose, Middlebrook 7H9 supplemented with 0.03% casitone, 0.4% glucose, and 0.05% tyloxapol. Cultures grown in this medium were diluted 1:500 prior to inoculation of the MIC assay. The new compounds **1**–**7** were reconstituted in DMSO to a concentration of 10 mM and 2-fold serial dilution duplicates were prepared across a 96-well microtiter plate, in a volume of 50 µL. Diluted *M. tuberculosis* cultures (50 µL) were added to each well of the plate (including control wells) to obtain a final volume of 100 µL. Rifampicin (at 2 × MIC) was used as assay control at a minimum growth control (rifampicin), and maximum growth control (5% DMSO). The microtiter plates were sealed in a secondary container and incubated at 37 °C with 5% CO_2 and humidification. Alamar Blue reagent was added to each well of the assay plate 24 h prior to fluorescence reading. Relative fluorescence (excitation at 485 nm; emission 520 nm) was measured using a plate reader (FLUOstar OPTIMA, BMG LABTECH) at day 14. The raw fluorescence data was archived and analyzed using the CDD Vault from Collaborative Drug Discovery, in which data was normalized to the minimum and maximum inhibition controls to generate a dose–response curve (percentage inhibition), using the Levenberg–Marquardt damped least squares method, from which the MIC was calculated [3]. The lowest concentration of the drug that inhibited growth by more than 90% of the mycobacterial population was considered the MIC.

Table S8. Antimicrobial activities (MIC in µg/ mL) of compounds **1–7**

**Compound ID**	**MRSA**	**MSSA**	***E.coli***	***A.b***	***E.f (VanA)***	***E. f (VS)***	***T. B***
**1**	>64	>64	>64	>64	>64	>64	>125
**2**	>64	>64	>64	>64	>64	>64	7.8
**3**	>64	>64	>64	>64	>64	16	15.6
**4**	>64	>64	>64	>64	>64	8-16	>125
**5**	>64	>64	>64	>64	>64	32	125
**6**	>64	>64	>64	>64	>64	>64	>64
**7**	>64	>64	>64	>64	>64	>64	>64
**Rifampicin**	-	-	-	-	-	-	0.02
**Vancomycin**	1-2	2-4	-	-	>128	8	-
**Aztreonam**	-	-	0.25-0.5	8	-	-	-

A.b *(A. baumannii), E.f* VanA (vancomycin-resistant *E. faecium VanA*15167) and *E.f VS* (Vancomycin-sensitive *E. faecium* VS144754) and *T.B* (*mycobacterium tuberculosis*).

References

(1) Jorgensen, J. H.; Carroll, K. C.; Guido F.; Pfaller, M. A.; Landry, M. L.; Richter, S. S.; Warnnock, D. *Clin. Microbiol.* (eleventh ed.), *ASM* **2015**, 1253-1273. doi:10.1086/686849

(2) Ioerger, T. R.; Feng, Y.; Ganesula, K.; Chen, X.; Dobos, K. M.; Fortune, S.; Jacobs, W. R.; Mizrahi, V.; Parish, T.; Rubin, E.; Sassetti, C.; Sacchettini, J. C. *J. Bacteriol.* **2010**, *192*, 3645-3653. doi:10.1128/jb.00166-10

(3) Tang, Y. J.; Shui, W.; Myers, S.; Feng, X.; Bertozzi, C.; Keasling, J. D. *Biotechnol. Lett.* **2009**, *31*, 1233-1240. doi:10.1007/s10529-009-9991-7
